# Insulin synthesized in the paraventricular nucleus of the hypothalamus regulates pituitary growth hormone production

**DOI:** 10.1172/jci.insight.135412

**Published:** 2020-08-20

**Authors:** Jaemeun Lee, Kyungchan Kim, Jae Hyun Cho, Jin Young Bae, Timothy P. O’Leary, James D. Johnson, Yong Chul Bae, Eun-Kyoung Kim

**Affiliations:** 1 Department of Brain and Cognitive Sciences, Daegu Gyeongbuk Institute of Science and Technology, Daegu, South Korea.; 2 Department of Oral Anatomy and Neurobiology, School of Dentistry, Kyungpook National University, Daegu, South Korea.; 3 Diabetes Research Group, Life Sciences Institute, Department of Cellular and Physiological Sciences, Faculty of Medicine, University of British Columbia, Vancouver, British Columbia, Canada.; 4 Neurometabolomics Research Center, Daegu Gyeongbuk Institute of Science and Technology, Daegu, South Korea.

**Keywords:** Endocrinology, Neuroscience, Insulin, Neuroendocrine regulation

## Abstract

Evidence has mounted that insulin can be synthesized in various brain regions, including the hypothalamus. However, the distribution and functions of insulin-expressing cells in the hypothalamus remain elusive. Herein, we show that in the mouse hypothalamus, the perikarya of insulin-positive neurons are located in the paraventricular nucleus (PVN) and their axons project to the median eminence; these findings define parvocellular neurosecretory PVN insulin neurons. Contrary to corticotropin-releasing hormone expression, insulin expression in the PVN was inhibited by restraint stress (RS) in both adult and young mice. Acute RS–induced inhibition of PVN insulin expression in adult mice decreased both pituitary growth hormone (*Gh*) mRNA level and serum GH concentration, which were attenuated by overexpression of PVN insulin. Notably, PVN insulin knockdown or chronic RS in young mice hindered normal growth via the downregulation of GH gene expression and secretion, whereas PVN insulin overexpression in young mice prevented chronic RS–induced growth retardation by elevating GH production. Our results suggest that in both normal and stressful conditions, insulin synthesized in the parvocellular PVN neurons plays an important role in the regulation of pituitary GH production and body length, unveiling a physiological function of brain-derived insulin.

## Abstract 2

Insulin produced in the paraventricular nucleus regulates body length by modulating pituitary growth hormone expression and secretion under both normal and stress conditions.

## Graphical abstract

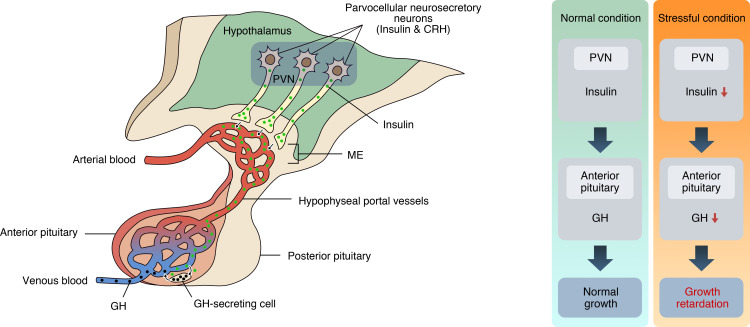

## Introduction

Insulin secreted from pancreatic β cells can cross the blood-brain barrier and therefore dominates the overall insulin content in the brain (1). Surprisingly, however, insulin expression has been also discovered in various brain regions such as the choroid plexus, olfactory bulb, cerebellum, cerebral cortex, hippocampus, and hypothalamus (2–6). A single-cell quantitative reverse transcription–polymerase chain reaction (qRT-PCR) study demonstrated that insulin 2 (*Ins2*) mRNA is present in GABAergic neurogliaform cells in the cerebral cortex of the rat (7). In situ hybridization and immunohistochemistry studies revealed that insulin is produced in the CA1 and CA3 pyramidal neurons and dentate granule neurons in the adult mammalian hippocampus (8–10). These findings suggest that local synthesis might be an alternative source of brain insulin, but the physiological role of brain-derived insulin has not been clarified.

Although several brain regions have been suggested to possess the capacity for insulin production, the existence of insulin-expressing cells in the hypothalamus remains controversial. *Insulin* mRNA of rodents exists in 2 nonallelic forms, *Ins1* and *Ins2*, and only *Ins2* is expressed in the brain (5, 11, 12). Li et al. claimed that the *Ins2* promoter is inactive in the hypothalamus of *Ins2*-Cre–knockin mice (13). On the contrary, Mehran et al. observed *Ins2* mRNA expression in the hypothalamus of *Ins2*^+/+^ mice, which was significantly higher than the background level seen in *Ins2*^−/−^ hypothalamus, as well as patterns of histone methylation that differed from those seen in islets (5). In terms of the mechanisms regulating insulin gene expression in the mouse hypothalamus, glucose was found to upregulate *Ins2* mRNA levels, while glucagon-like peptide 1 and forskolin biphasically regulated *Ins2* mRNA levels in a mouse hypothalamic cell line in a time-dependent manner (14). We have previously reported that acute Wnt3a injection into the third ventricle of the mouse brain increases hypothalamic *Ins2* mRNA levels by triggering the expression of NeuroD1, which is a transcription factor for insulin (15). These findings reinforce the notion that insulin-expressing cells exist within the hypothalamus, but the regional distribution of the populations of these cells has not yet been sufficiently explored.

The hypothalamus consists of several subregions with different nuclei, including the paraventricular nucleus (PVN). The PVN consists of 3 types of neurons: parvocellular neurosecretory, parvocellular pre-autonomic, and magnocellular neurosecretory neurons. The parvocellular neurosecretory neurons produce several neurohormones, including thyrotropin-releasing hormone (TRH), corticotropin-releasing hormone (CRH), and somatostatin (SST), and release these neurohormones through their neurosecretory nerve terminals in the external zone of the median eminence (ME) (16–18). The neurohormones are then transported to the anterior pituitary via hypophyseal portal vessels adjacent to the ME and regulate the production of a wide array of anterior pituitary hormones, including growth hormone (GH) (16, 19–21). The hypothalamo–pituitary regulation mediates the body’s responses to physiological stimuli or challenges, especially stress, by regulating hormone levels (22, 23).

In this study, we identify potentially novel parvocellular neurosecretory neurons as the PVN insulin-expressing neurons. We discovered a physiological role of PVN insulin in regulating pituitary GH gene expression and secretion, leading to a change in body length of young mice under both normal and stress conditions. This study will help better understanding of the regulatory roles of brain-derived insulin in hormone production, growth, and other bodily functions.

## Results

### Hypothalamic insulin is synthesized mainly in the PVN neurons.

**Figure 1:**
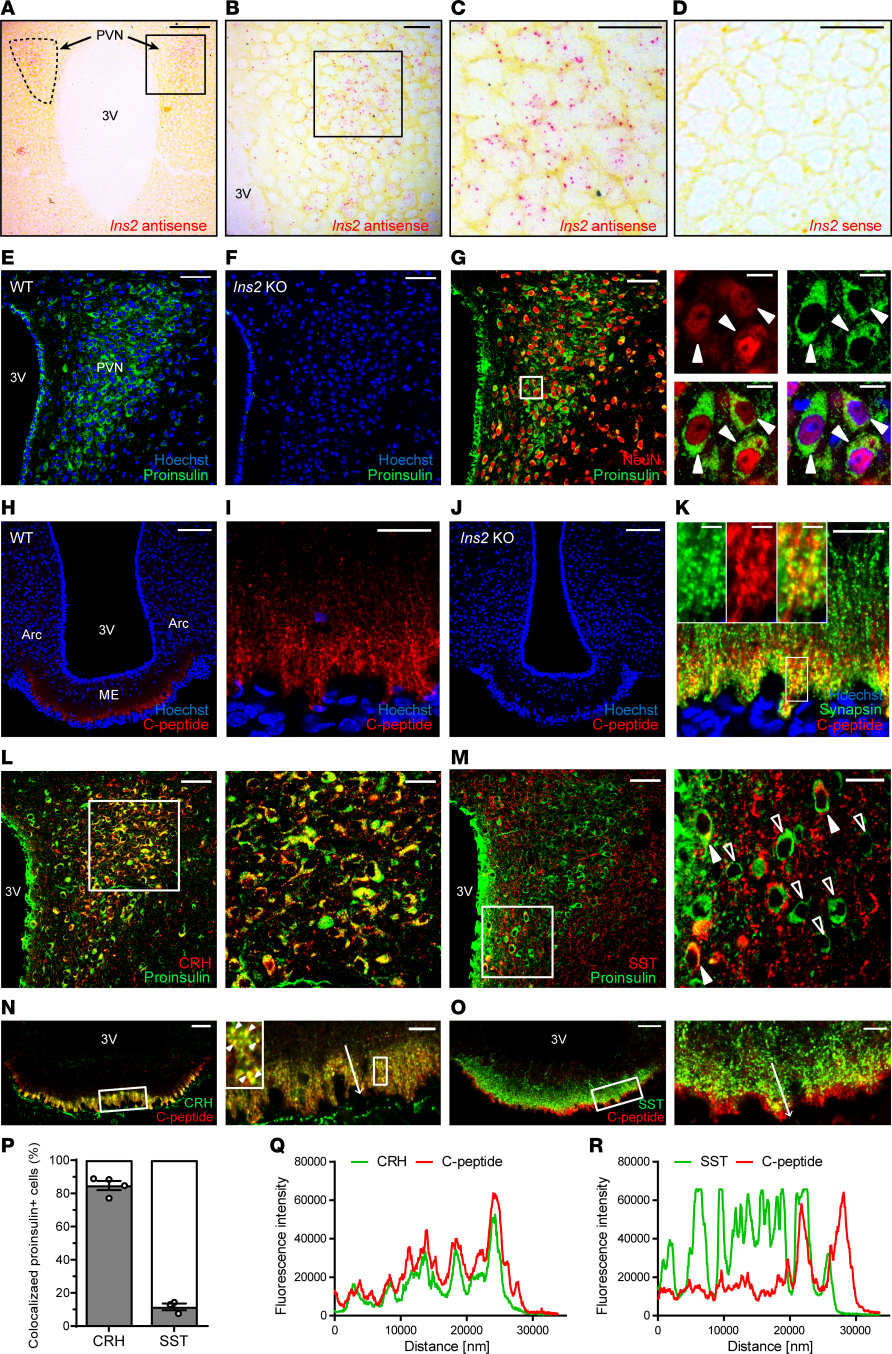
Proinsulin and C-peptide in the hypothalamus are localized in the PVN neuronal somata and ME neurosecretory nerve terminals, respectively. (**A**–**D**) In situ hybridization images. PVN sections were incubated with the *Ins2* antisense probe (**A**, low magnification; **B** and **C**, high magnification) or with the *Ins2* sense probe (**D**). (**E** and **F**) An immunofluorescence image showing the presence of proinsulin immunoreactivity in the PVN of WT mice (**E**). The specificity of the proinsulin antibody (R&D Systems, Bio-Techne, MAB13361) was validated in the PVN sections from *Ins2*-KO mice (**F**). (**G**) A confocal image of double immunostaining for proinsulin and the neuronal marker NeuN in the PVN. Solid arrowheads show colocalization. (**H**–**J**) Immunofluorescence images showing the presence of C-peptide immunoreactivity in the ME of WT mice (**H**, low magnification; **I**, high magnification). The specificity of the C-peptide antibody (Cell Signaling Technology, 4593) was validated in the ME sections from *Ins2*-KO mice (**J**). (**K**) A confocal image of double immunostaining for C-peptide and the presynaptic marker synapsin in the ME. (**L** and **M**) Confocal images of double immunostaining of proinsulin with CRH (**L**) or SST (**M**) in the PVN. Open arrowheads indicate single proinsulin labeling. Solid arrowheads show colocalization. (**N** and **O**) Confocal images of double immunostaining of C-peptide with CRH (**N**) or SST (**O**) in the ME. Solid arrowheads indicate colocalization. (**P**) The percentage of proinsulin-positive cells coexpressing CRH or SST in the PVN. (**Q** and **R**) Fluorescence intensity of C-peptide and CRH (**Q**) or SST (**R**) along white arrows in the enlarged images in (**N**) and (**O**), respectively. Data are shown as mean ± SEM, *n* = 3 or 4 mice/group. Scale bars: 200 μm (**A**), 100 μm (**H** and **J**), 50 μm (**E**–**G** and **L**–**O**), 20 μm (**B**–**D**, **I**, and **K**; high-magnification inset images in **L**–**O**), 10 μm (high-magnification inset images in **G**), 5 μm (high-magnification inset images in **K**). 3V, third ventricle; PVN, paraventricular nucleus; Arc, arcuate nucleus; ME, median eminence.

To determine which hypothalamic subregions express insulin, we first conducted in situ hybridization on serial coronal sections of the hypothalamus. Clear *Ins2* mRNA*–*positive signals were observed in PVN sections incubated with the *Ins2* antisense probe (Figure 1, A–C; and Supplemental Figure 1A; supplemental material available online with this article; https://doi.org/10.1172/jci.insight.135412DS1) but not with the sense probe (Figure 1D). Consistent with previous studies (8, 10), *Ins2* mRNA expression was also found in the hippocampal granule cell layer (Supplemental Figure 1B). Furthermore, immunostaining using an antibody specific for proinsulin, a biosynthetic precursor of insulin, showed that insulin-expressing cells were clearly located within the PVN (Figure 1E). Immunostaining using another antibody that recognizes both proinsulin and mature insulin also showed that insulin-expressing cells existed in the PVN (Supplemental Figure 1C). PVN sections from *Ins1*^+/+^ *Ins2*^−/−^ (*Ins2*-KO) mice showed no immunoreactivity to each antibody (Figure 1F and Supplemental Figure 1D), supporting not only the specificity of the antibodies but also the expression of PVN insulin. The specificity of the antibodies was further validated with the pancreas sections of WT and *Ins2*-KO mice (Supplemental Figure 2, A and B). To verify the colocalization of *Ins2* mRNA and proinsulin protein within the same PVN cells, we next monitored β-gal expression in the nucleus as a surrogate marker of *Ins2* gene expression because the coding sequencing of the *Ins2* gene was replaced with *LacZ* gene in the *Ins2*-KO mice used in our study (5). As expected, all proinsulin-positive cells contained β-gal–positive nuclei in the PVN sections from *Ins1*^+/+^ *Ins2*^+/−(β-gal)^ (*Ins2*^+/β-gal^) mice (Supplemental Figure 3). Last, immunoblot analysis detected proinsulin-positive bands in the hypothalamus and microdissected PVN of WT mice but not in *Ins2*-KO mice (Supplemental Figure 4). Collectively, these data demonstrate that insulin-expressing cells are present in the hypothalamic PVN.

To identify the cell types that express insulin in the PVN, we performed double immunostaining of proinsulin with NeuN (a neuronal nuclear marker), microtubule-associated protein 2 (MAP2; a neuronal soma and dendrite marker), glial fibrillary acidic protein (GFAP; an astrocyte marker), or Iba-1 (a microglial marker). Proinsulin-positive cells in the PVN contained mainly NeuN-positive nuclei (Figure 1G). Costaining with MAP2 revealed that proinsulin was prominently expressed in neuronal somata rather than dendrites in the PVN (Supplemental Figure 1E). Proinsulin was not colocalized with GFAP-positive astrocytes in the PVN (Supplemental Figure 1F). However, we found that proinsulin-positive cells overlapped with GFAP-expressing ependymal cells lining the third ventricle (Supplemental Figure 1F). In the PVN, proinsulin immunoreactivity was also detected in amoeboid microglia but not in ramified microglia, although the microglial population with amoeboid morphology was minor (Supplemental Figure 1G). Taken together, these data show that insulin is expressed predominantly in neurons rather than astrocytes or microglia in the PVN and is located specifically in the neuronal somata.

To validate both de novo insulin synthesis and processing in the hypothalamus, we also performed immunostaining for C-peptide, which is a small peptide that connects the A and B chains of proinsulin and is excised during proinsulin processing and thus is a proxy for processed insulin. No C-peptide–positive signals were observed in the PVN (Supplemental Figure 1H). Instead, C-peptide immunoreactivity was detected exclusively in the ME of the hypothalamus (Figure 1H). A high-magnification image revealed that C-peptide was present in a diffuse punctate pattern within the external zone of the ME (Figure 1I). ME sections from *Ins2*-KO mice showed no immunoreactivity for C-peptide (Figure 1J). The specificity of the C-peptide antibody was validated in the pancreas sections of WT and *Ins2*-KO mice (Supplemental Figure 2, A and B). Proinsulin-positive signals were not evident in the external zone of the ME (Supplemental Figure 1I).

The ME contains neurosecretory nerve terminals, tanycytes, astrocytes, and microglia (24, 25). To investigate the origin of C-peptide present in the ME, we performed double immunostaining of C-peptide with synapsin (a presynaptic marker), vimentin (a tanycyte marker), GFAP, or Iba-1. C-peptide was highly colocalized with synapsin-positive presynaptic nerve terminals in the external zone of the ME (Figure 1K). In contrast, C-peptide was not colocalized with vimentin, although they were closely juxtaposed along the external zone of the ME (Supplemental Figure 1J). C-peptide did not overlap with GFAP or Iba-1 in the ME (Supplemental Figure 1, K and L). These data indicate that C-peptide in the ME is constitutively situated in neurosecretory nerve terminals but not in tanycytes, astrocytes, or microglia.

### A subset of PVN insulin neurons coexpress CRH or SST.

To investigate whether PVN insulin neurons also contain other neurohormones typically produced by parvocellular neurosecretory neurons, we performed double immunostaining of proinsulin with CRH or SST in the PVN and of C-peptide with CRH or SST in the ME. For these experiments, we used a CRH or SST antibody that recognizes both the precursor and mature form. Most proinsulin-positive cells contained CRH in the PVN (84.78% ± 2.73%, Figure 1, L and P). Inversely, 97.078% ± 0.47% of CRH-positive cells were immunoreactive for proinsulin (data not shown). C-peptide–positive nerve terminals were highly colocalized with CRH-positive nerve terminals in the ME (Figure 1, N and Q). Some proinsulin-positive cells also contained SST, especially in the periventricular regions of the PVN (11.58% ± 2.07%, Figure 1, M and P). A small portion of C-peptide–positive nerve terminals were colocalized with SST-positive nerve terminals in the ME, as indicated by rare yellow puncta (Figure 1, O and R). These data suggest that subpopulations of PVN insulin neurons produce and transport CRH or SST along with insulin.

### PVN insulin neurons send their nerve terminals to the external zone of the ME.

**Figure 2:**
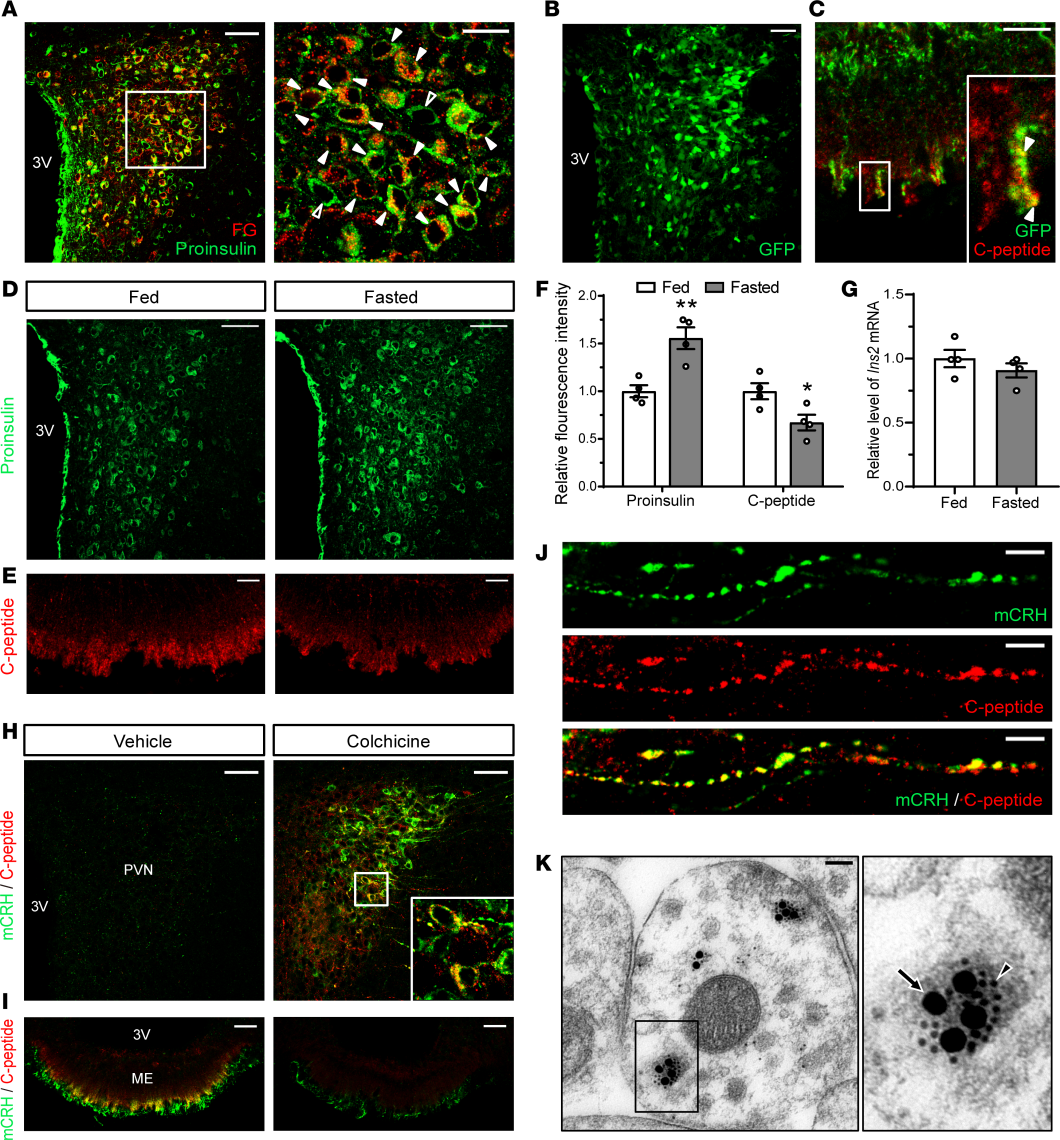
PVN insulin neurons project to the external zone of the ME, thereby cotransporting insulin and CRH from the PVN to the ME. (**A**) A confocal image of double immunostaining for proinsulin and FG in the PVN of mice i.p. injected with FG. Open arrowheads indicate single proinsulin labeling. Solid arrowheads show colocalization. (**B** and **C**) Taken 2 weeks after injection of GFP-expressing lentivirus into the PVN, a confocal image of GFP expression in the PVN (**B**) and of double immunostaining for C-peptide and GFP in the ME (**C**). Solid arrowheads show colocalization. (**D**–**G**) Confocal images of the PVN and ME from fed and 24-hour fasted mice and qRT-PCR analysis of *Ins2* mRNA levels in the microdissected PVN. The PVN was immunostained for proinsulin (**D**) and the ME for C-peptide (**E**). Quantification of fluorescence intensity of proinsulin-immunoreactive cells in the PVN and C-peptide–immunoreactive nerve terminals in the ME (**F**). Relative levels of *Ins2* mRNA in the PVN (**G**). (**H**–**J**) Confocal images of double immunostaining for mCRH and C-peptide 48 hours after vehicle or colchicine injection. PVN (**H**) and ME (**I**) at low magnification (**H**). PVN at high magnification (**J**). (**K**) Double immunogold labeling showing colocalization of mCRH and C-peptide within the same neurosecretory granules in an axon nerve terminal located in the ME. An arrowhead indicates an mCRH-immunoreactive 12 nm gold particle. An arrow denotes a C-peptide–immunoreactive 30 nm gold particle. Data are shown as mean ± SEM, 2-tailed unpaired Student’s *t* test. ** *P* < 0.01, *n* = 4 mice/group (**F**), *n* = 6 mice/group (**G**). Scale bars: 50 μm (**A**, **B**, **D**, **H**, and **I**), 20 μm (**C**, **E**, and high-magnification inset images in **A**), 10 μm (**J**), 100 nm (**K**).

Considering that parvocellular neurosecretory neurons of the PVN send their nerve terminals into the external zone of the ME (26, 27), we intraperitoneally injected Fluorogold (FG), a retrograde axonal tracer, to examine whether the axons of PVN insulin neurons terminate in the ME. FG circulating in the blood is taken up by nerve terminals lying outside the blood-brain barrier and is retrogradely transported to neuronal somata, resulting in the labeling of neurons projecting to the external zone of the ME (28–30). Five days after i.p. injection of FG, the vast majority of PVN insulin neurons contained FG (Figure 2A), indicating that these neurons have the anatomical properties of parvocellular neurosecretory neurons. Conversely, to ascertain whether C-peptide in the ME comes from parvocellular neurosecretory neurons, we performed anterograde tracing experiments using lentivirus expressing GFP. Injection of the lentivirus into the PVN yielded high GFP expression within the PVN (Figure 2B). Two weeks after the injection, GFP-labeled nerve terminals of parvocellular neurosecretory neurons overlapped with C-peptide–positive nerve terminals in the external zone of the ME (Figure 2C), suggesting that some parvocellular neurosecretory neurons deliver C-peptide into the ME along their axons. Several hypothalamic neuropeptides, such as TRH and CRH, show different localization between premature and mature forms (31, 32). This is because mature neuropeptides produced by processing of immature proteins in the neuronal somata are rapidly delivered to axon terminals through axonal transport (33). Because the colocalization of proinsulin and C-peptide was not observed in the normal PVN, we next administered colchicine, a blocker of rapid axonal transport, into the left lateral ventricle, to block axonal transport of C-peptide from the PVN to the ME and examine whether the colchicine injection leads to colocalization of proinsulin and C-peptide in the PVN. C-peptide–positive signals were not apparent in the PVN of vehicle-injected mice but were clearly observed in cell bodies and axon-like processes and perfectly colocalized with proinsulin-positive cell bodies in the PVN after the colchicine injection (Supplemental Figure 5A). Conversely, a dramatic reduction in C-peptide–positive nerve terminals was observed in the ME of colchicine-injected mice compared with that of vehicle-injected mice (Supplemental Figure 5B). These data demonstrate that C-peptide is transported from the somata of insulin-expressing neurons in the PVN to their axon terminals in the ME, suggesting that PVN insulin neurons transport insulin to the ME through axonal projections.

Several members of the prohormone convertase (PC) family are distributed throughout the PVN (34). Among them, the enzymatic activities of PC1 and PC2 in the parvocellular PVN neurons are remarkably diminished following starvation (31). Because PC1 and PC2 convert proinsulin into insulin and C-peptide within secretory granules (35), we hypothesized that fasting induces the accumulation of proinsulin in the PVN by slowing its proteolytic processing. The fluorescence intensity of proinsulin was significantly higher in the PVNs of fasted mice than in fed controls (Figure 2, D and F). In contrast, the fluorescence intensity of C-peptide was significantly lower in the ME of fasted mice than in that of fed controls (Figure 2, E and F). However, there was no significant difference in *Ins2* mRNA levels in the PVN between fed and fasted mice (Figure 2G). These data further confirm that insulin is transported from the PVN to the ME.

### PVN neurons expressing both insulin and CRH cotransport these proteins into the ME.

To examine whether CRH synthesized in the PVN insulin neurons is transported into the ME along with insulin, we conducted double immunostaining for CRH and C-peptide in colchicine-injected mice. In this experiment, we used a CRH antibody that detects mature CRH (mCRH) but not the CRH precursor (32). Neither mCRH-positive nor C-peptide–positive cell bodies were detected in the PVN of vehicle-injected mice, but both proteins were strongly colocalized in the PVN of colchicine-injected mice (Figure 2H). A noticeable overlap was found between mCRH-positive and C-peptide–positive nerve terminals in the ME of vehicle-injected mice, but their fluorescence signals were conspicuously diminished in the ME of colchicine-injected mice (Figure 2I). Similarly, axon collaterals positive for both mCRH and C-peptide were found in the PVN of colchicine-injected mice (Figure 2J). Last, electron microscopy analysis with immunogold staining showed that mCRH and C-peptide were extensively colocalized in the same large dense-core vesicles within axon terminals in the ME of normal mice (Figure 2K). Taken together, these data indicate that PVN neurons coexpressing insulin and CRH copackage insulin and mCRH in the same neurosecretory granules and transport them to the ME.

### Acute restraint stress inhibits PVN insulin expression.

**Figure 3:**
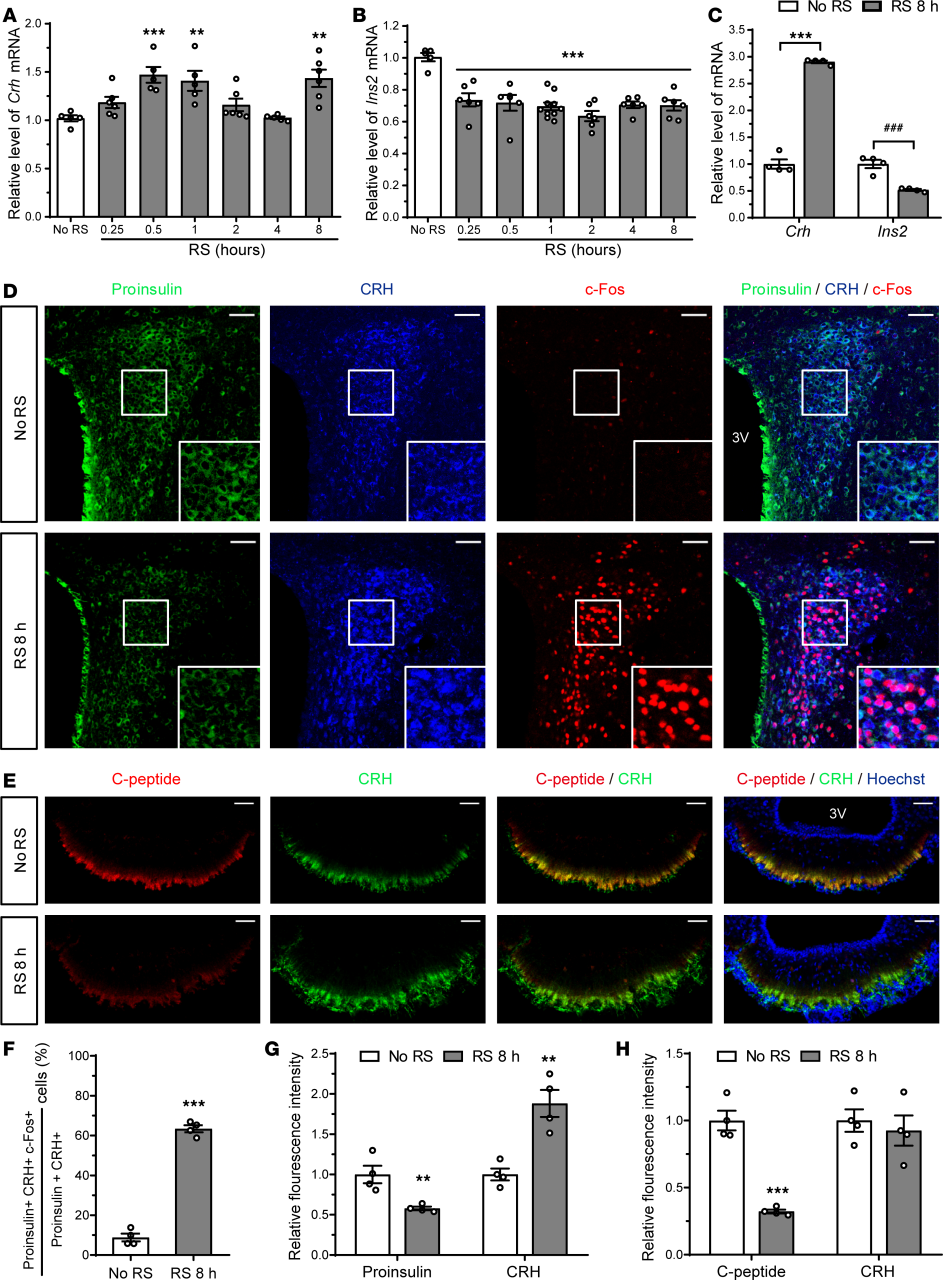
Insulin synthesis in the PVN is suppressed by acute RS. (**A** and **B**) Time course study of hypothalamic *Crh* (**A**) and *Ins2* (**B**) mRNA expression after acute RS from 15 minutes to 8 hours. Data are shown as mean ± SEM, 1-way ANOVA with Bonferroni’s post hoc tests. **P < 0.01, ***P < 0.001, *n* = 5–7 mice/group. (**C**) Effects of acute RS for 8 hours (RS 8 h) on the relative levels of *Crh* and *Ins2* mRNA in the microdissected PVN. (**D**–**H**) Confocal images of the PVN and ME and quantitative analysis of the positive cell number and fluorescence intensity. PVN sections triple immunostained for proinsulin, CRH, and the neuronal activity marker c-Fos (**D**). ME sections double immunostained for C-peptide and CRH (**E**). The percentage of proinsulin^+^CRH^+^ cells coexpressing c-Fos (**F**). Fluorescence intensity of proinsulin- or CRH-immunoreactive cells in the PVN (**G**) and of C-peptide– or CRH-immunoreactive nerve terminals in the ME (**H**). Data are shown as mean ± SEM, 2-tailed unpaired Student’s *t* test. **P < 0.01, *** and ^###^*P* < 0.001, *n* = 9 mice/group (**C**), *n* = 4 mice/group (**F**–**H**). Scale bars: 50 μm. No RS, no restraint stress group; RS, restraint stress group.

CRH is the principal factor mediating stress responses, and PVN CRH neurons rapidly produce CRH upon exposure to stress (22, 23). In this regard, we wondered how PVN insulin neurons respond to stress. First, we analyzed the time course of hypothalamic CRH and insulin gene expression during acute restraint stress (RS) for 8 hours. In line with previous reports (36, 37), hypothalamic *Crh* mRNA levels were elevated by acute RS, displaying a biphasic response with peaks at 30 minutes and 8 hours (Figure 3A). However, hypothalamic *Ins2* mRNA levels were steadily downregulated at all time points of RS (Figure 3B). Likewise, in the microdissected PVNs of mice exposed to RS for 8 hours, *Crh* mRNA levels were increased, whereas *Ins2* mRNA levels were decreased (Figure 3C). Next, we performed triple immunostaining for proinsulin, CRH, and the neuronal activity marker c-Fos in the PVN and double immunostaining for C-peptide and CRH in the ME using brain sections from mice subjected to RS for 8 hours. Acute RS significantly increased the percentage of proinsulin/CRH co-positive cells containing c-Fos compared with control mice (Figure 3, D and F), indicating that PVN neurons coexpressing insulin and CRH were activated by acute RS. The fluorescence intensity of proinsulin in the PVN was significantly lower in acute-RS mice than in control mice (Figure 3, D and G). The fluorescence intensity of C-peptide in the ME was also significantly lower in acute-RS mice than in control mice (Figure 3, E and H). In contrast, the fluorescence intensity of CRH in the PVN was significantly higher in acute-RS mice than in control mice (Figure 3, D and G). However, the fluorescence intensity of CRH in the ME was similar between control and acute-RS mice (Figure 3, E and H), despite a remarkable increase in CRH synthesis in the PVN, similar to previous studies (38, 39). Collectively, these data suggest that insulin expression, unlike that of CRH, is consistently inhibited in the PVN under acute-RS conditions.

### PVN insulin supports pituitary GH production.

**Figure 4:**
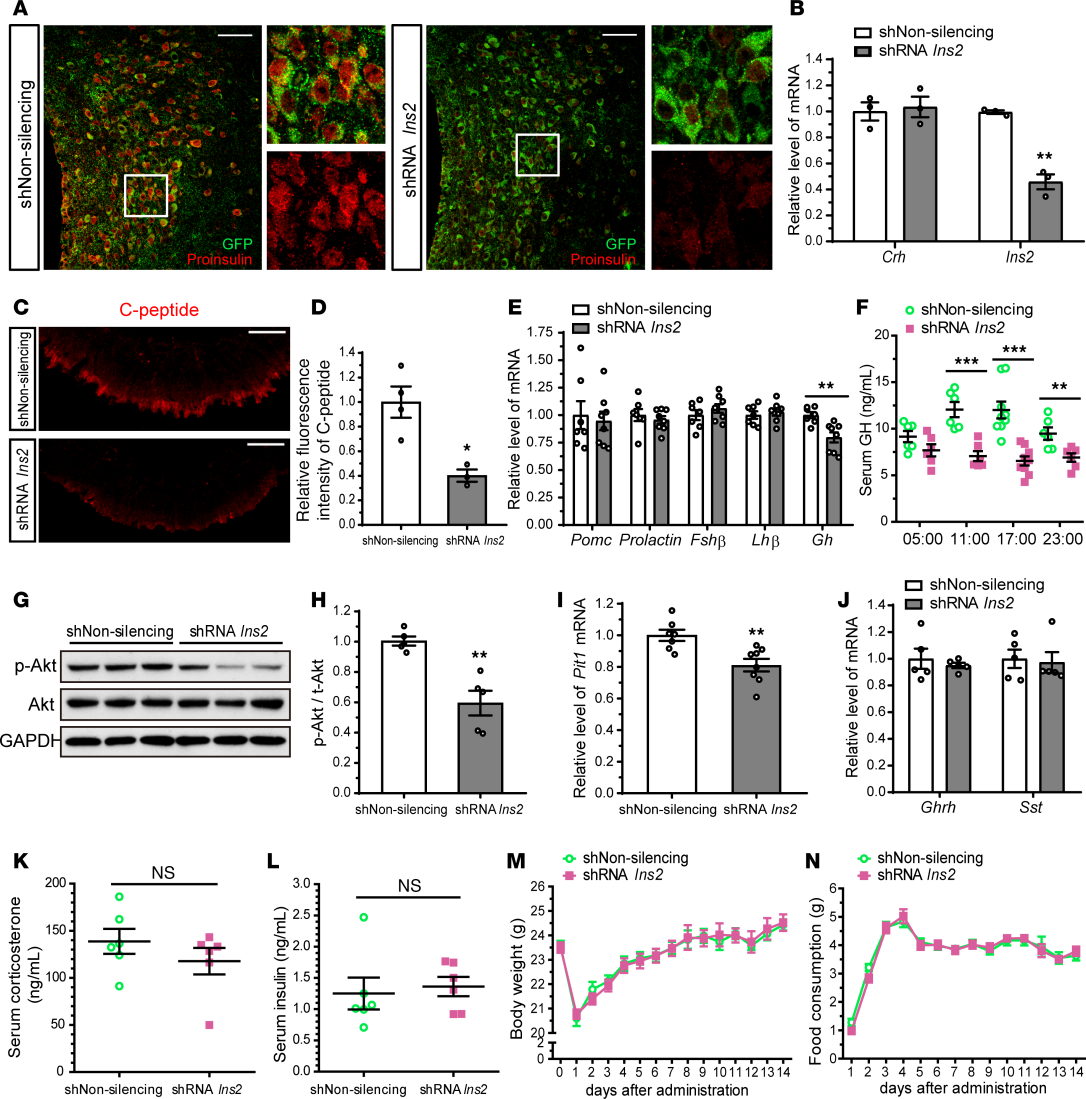
Insulin knockdown in the PVN of adult mice reduces pituitary GH gene expression and secretion. GFP lentiviral vector carrying nonsilencing shRNA control (shNon-silencing) or *Ins2* shRNA (shRNA *Ins2*) was injected into the PVN of adult mice. (**A**) A confocal image showing colocalization of the GFP-infected cells with proinsulin in the PVN 2 weeks after the lentiviral injection. (**B**) qRT-PCR data showing the efficient knockdown of *Ins2*, but not *Crh*, by *Ins2* shRNA in the microdissected PVN. (**C** and **D**) Confocal images of C-peptide–immunoreactive nerve terminals in the ME (**C**) and quantification of the fluorescence intensity (**D**). (**E**) Relative levels of the mRNA for anterior pituitary hormones in the pituitary. (**F**) Serum GH concentrations in tail vein samples taken 4 times a day. (**G** and **H**) Immunoblot analysis of p-Akt (Ser473), Akt, and GAPDH levels in the pituitary (**G**). Quantification of p-Akt levels normalized to Akt (**H**). (**I**) Relative pituitary *Pit-1* mRNA levels. (**J**) Relative hypothalamic GH–releasing hormone (*Ghrh*) or *Sst* mRNA levels. (**K** and **L**) Serum corticosterone (**K**) and insulin (**L**) concentrations. (**M** and **N**) Changes in body weight (**M**) and food consumption (**N**) for 2 weeks after the injection. Data are shown as mean ± SEM, 2-tailed unpaired Student’s *t* test. *P < 0.05, **P < 0.01, ***P < 0.001, *n* = 6–9 mice/group (**B**, **E**, **F**, and **I**–**N**), *n* = 3 or 4 mice/group (**D**), *n* = 5 mice/group (**H** and **J**). Scale bars: 50 μm.

Neurohormones synthesized in parvocellular neurosecretory neurons ultimately regulate the production of a variety of anterior pituitary hormones, including GH (16, 19–21). This raises the intriguing possibility that insulin produced in PVN neurons may control the gene expression and secretion of anterior pituitary hormones. To examine this possibility, we knocked down the insulin gene in the PVN of adult mice (8 weeks old). Two weeks after injection of GFP lentivirus carrying 4 different *Ins2* shRNA constructs into the PVN, successful knockdown of insulin in the PVN was shown by reduced proinsulin immunoreactivity in GFP-positive cells (Figure 4A) and a significant decrease in PVN *Ins2* mRNA level (Figure 4B), in comparison with nonsilencing shRNA–injected control mice. C-peptide immunoreactivity was also significantly lower in the ME of *Ins2* shRNA–injected mice than in that of control mice (Figure 4, C and D). *Crh* mRNA levels in the PVN were not altered in *Ins2* shRNA–injected mice (Figure 4B). Next, we analyzed the mRNA levels of several anterior pituitary hormones. Interestingly, of these hormones, only *Gh* mRNA expression was significantly downregulated by the knockdown of PVN insulin (Figure 4E). Because GH is released from the anterior pituitary in a pulsatile manner (40), serum GH levels were assessed from vein samples taken at 6-hour intervals throughout the day. Serum GH concentrations were also remarkably lower in PVN insulin–knockdown mice than in control mice (Figure 4F). These data suggest that PVN insulin contributes to sustained GH gene expression and secretion in the anterior pituitary.

GH-secreting cells of the pituitary express insulin receptors (41). However, it is still unclear whether a classic insulin signaling pathway mediates GH production. Intriguingly, PVN insulin knockdown significantly suppressed phosphorylation of Akt (p-Akt), a major target of insulin signaling, in the pituitary (Figure 4, G and H). Considering that pituitary-specific positive transcription factor 1 (Pit-1) is an essential transcription factor for GH (42–44), we also assessed *Pit-1* mRNA levels. *Pit-1* mRNA in the pituitary of PVN insulin–knockdown mice was significantly downregulated compared with that of control mice (Figure 4I).

Hypothalamic GHRH and SST are major GH regulators. Pituitary GH gene expression and secretion are induced by GHRH but inhibited by SST released from the hypothalamus (45–47). To investigate whether PVN insulin indirectly regulates pituitary GH gene expression and secretion by changing hypothalamic GHRH or SST production, we quantified *Ghrh* and *Sst* mRNA levels in the hypothalamus of PVN insulin–knockdown mice. Knockdown of PVN insulin did not affect hypothalamic *Ghrh* and *Sst* mRNA levels (Figure 4J). Considering that serum glucocorticoids and insulin modulate GH production (48), we also analyzed serum levels of corticosterone (a dominant glucocorticoid in rodents) and insulin but found no significant differences between control and PVN insulin–knockdown mice (Figure 4, K and L). There were no significant differences in body weight or food consumption between control and PVN insulin–knockdown mice (Figure 4, M and N).

### Acute RS–induced suppression of PVN insulin expression attenuates pituitary GH production.

**Figure 5:**
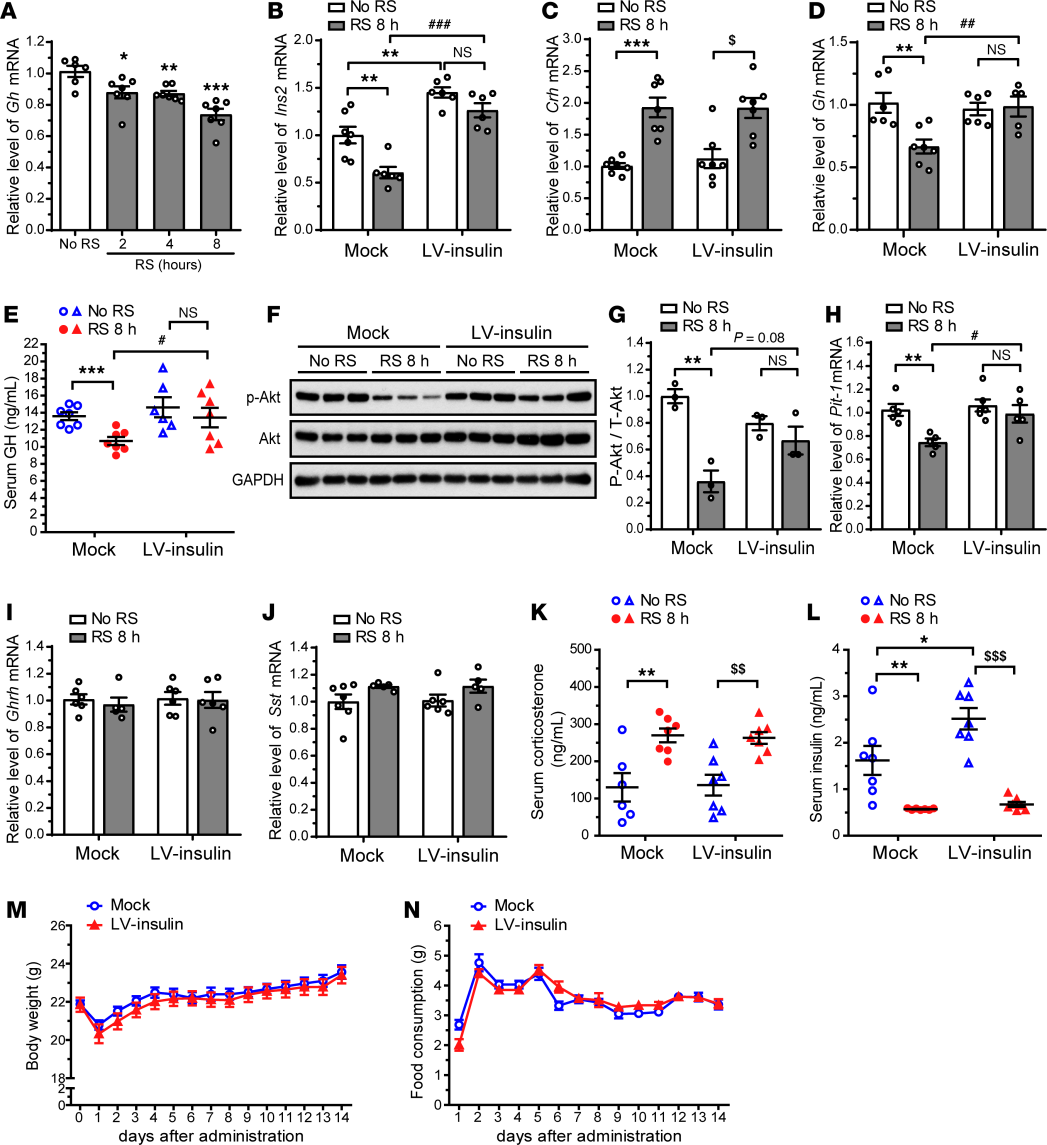
Insulin overexpression in the PVN of adult mice prevents acute RS–induced reduction in pituitary GH gene expression and secretion. (**A**) Time course study of *Gh* mRNA levels in the pituitary of adult mice after acute RS. (**B**–**L**) Lentiviral vector expressing GFP (empty control vector, mock) or GFP and *Ins2* (LV-insulin) was injected into the PVN of adult mice. Two weeks after the injection, mice were exposed to acute RS for 8 hours (RS 8 h). qRT-PCR data showing the efficiency of PVN insulin overexpression (**B**). Relative hypothalamic *Crh* mRNA levels (**C**). Relative pituitary *Gh* mRNA levels (**D**). Serum GH concentrations (**E**). Immunoblot analysis of p-Akt (Ser473), Akt, and GAPDH levels (**F**). Quantification of p-Akt levels normalized to Akt (**G**). Relative pituitary *Pit-1* mRNA levels (**H**). Relative hypothalamic *Ghrh* (**I**) and *Sst* (**J**) mRNA levels. Serum corticosterone (**K**) and insulin (**L**) concentrations. Changes in body weight (**M**) and food consumption (**N**) for 2 weeks after the injection. Data are shown as mean ± SEM, 2-way ANOVA with Bonferroni’s post hoc tests. *, ^#^, and ^$^*P* < 0.05; ** and ^##^*P* < 0.01; *** and ^###^*P* < 0.001, *n* = 5–7 mice/group (**A**–**E** and **H**–**L**), *n* = 3 mice/group (**G**), *n* = 14 mice/group (**M** and **N**).

Our results showed that knockdown of PVN insulin leads to downregulation of pituitary GH production. In addition, acute RS suppresses PVN insulin expression and concomitantly decreases pituitary *Gh* mRNA levels relative to control (no RS) in a time-dependent manner (Figure 5A). On the basis of these findings, we hypothesized that the suppression of PVN insulin expression during acute RS would decrease pituitary GH production. To test this hypothesis, we overexpressed insulin in the PVN of adult mice by injecting a lentivirus carrying *Ins2* into the PVN (LV-insulin) and examined whether overexpression of PVN insulin reverses acute RS–induced decrease in pituitary GH production. Two weeks after the injection, the *Ins2* mRNA level in the PVN was significantly higher in LV-insulin mice than in empty vector–injected control mice (mock) (Figure 5B). Acute RS–induced reduction in PVN *Ins2* mRNA levels was rescued by overexpression of insulin in the PVN (Figure 5B). Hypothalamic *Crh* mRNA levels were elevated following acute RS regardless of PVN insulin overexpression (Figure 5C). Of note, overexpression of insulin in the PVN abolished acute RS–induced reduction in pituitary *Gh* mRNA levels (Figure 5D) and blunted reductions in serum GH concentrations (Figure 5E). These data suggest that the acute RS–induced decline in PVN insulin levels reduces pituitary GH gene expression and secretion, whereas overexpression of insulin in the PVN prevents the acute RS–induced reduction in pituitary GH production.

Acute RS significantly reduced p-Akt levels in the pituitary of mock mice, but overexpression of insulin in the PVN attenuated the acute RS–induced reduction in p-Akt levels (Figure 5, F and G). *Pit-1* mRNA levels were not significantly reduced in the pituitary of LV-insulin mice, as opposed to mock mice, following acute RS (Figure 5H).

Hypothalamic *Ghrh* and *Sst* mRNA levels were unaffected by insulin overexpression in the PVN under either control or acute-RS conditions (Figure 5, I and J). Serum corticosterone level was markedly elevated in both control and LV-insulin mice following acute RS (Figure 5K). Although the serum insulin level was significantly higher in LV-insulin than in control mice, this difference was offset by a profound reduction in serum insulin following acute RS (Figure 5L). Body weight and food consumption did not show significant differences between control and LV-insulin mice (Figure 5, M and N).

### PVN insulin regulates body length by modulating pituitary GH production.

**Figure 6:**
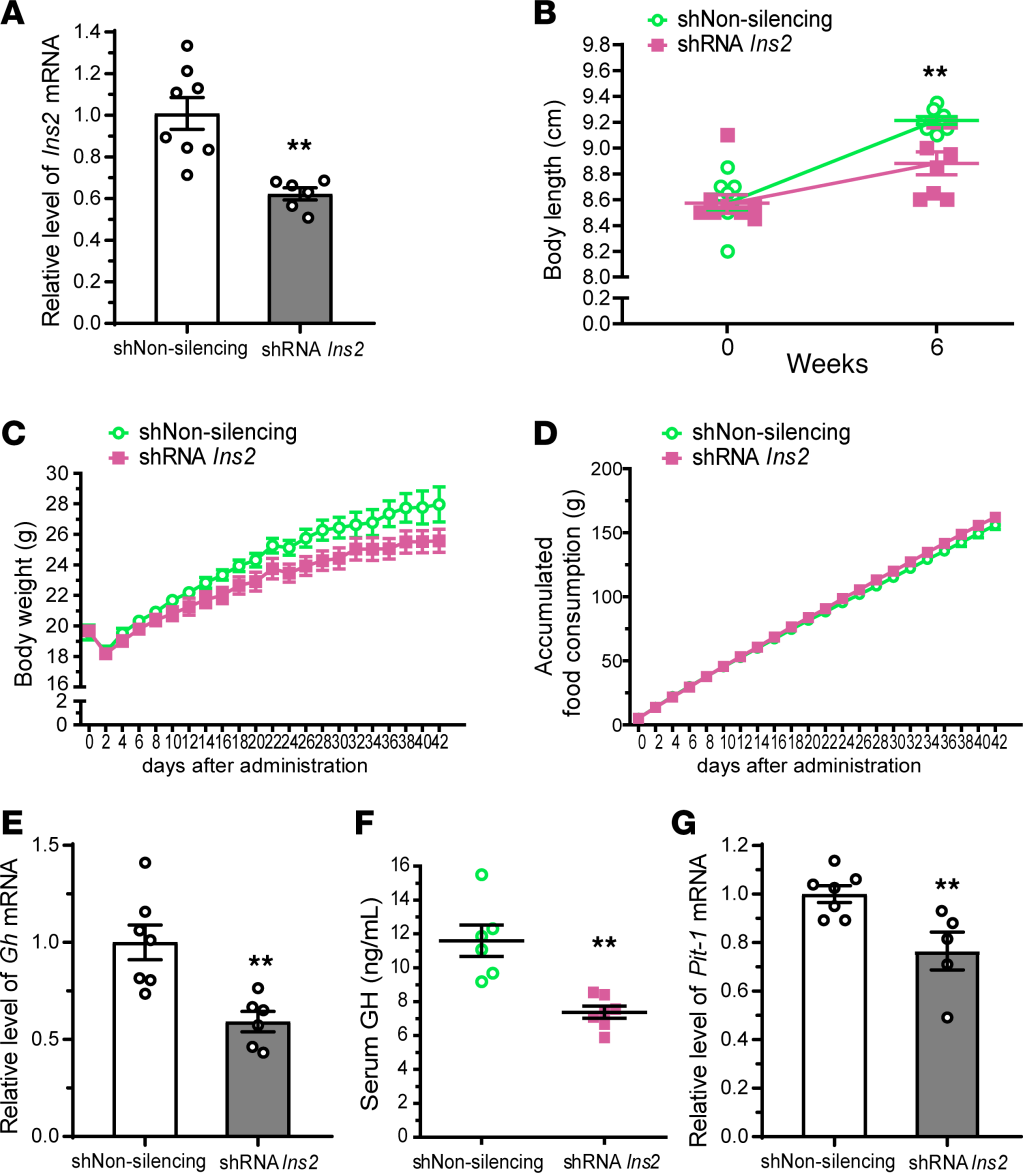
Insulin knockdown in the PVN of young mice causes growth retardation. Lentiviral vector carrying nonsilencing shRNA control (shNon-silencing) or *Ins2* shRNA (shRNA *Ins2*) was injected into the PVN of young mice. (**A**) Relative hypothalamic *Ins2* mRNA levels 6 weeks after the injection. (**B**) Body length from nose to rump immediately before the injection and 6 weeks after the injection. (**C** and **D**) Time course study of body weight (**C**) and accumulated food consumption (**D**). (**E**) Relative pituitary *Gh* mRNA levels. (**F**) Serum GH concentrations. (**G**) Relative pituitary *Pit-1* mRNA levels. Data are shown as mean ± SEM, 2-tailed unpaired Student’s *t* test. **P < 0.01, *n* = 6–8 mice/group.

GH promotes growth until adolescence in mammals (49–51). Therefore, to investigate the effect of PVN insulin on growth, we injected lentivirus containing *Ins2* shRNA into the PVN of young mice (4 weeks old). Six weeks after the injection, hypothalamic *Ins2* mRNA expression was significantly downregulated (Figure 6A), and interestingly, a significant reduction in body length was observed (Figure 6B) in PVN insulin–knockdown mice in comparison with nonsilencing shRNA–injected control mice. Although there were no significant differences in body weight or food consumption between control and PVN insulin–knockdown mice during the 6 weeks (Figure 6, C and D), the weight gain of PVN insulin–knockdown mice tended to be slightly slower than that of control mice (Figure 6C). Relative pituitary *Gh* mRNA levels (Figure 6E), serum GH concentrations (Figure 6F), and relative *Pit-1* mRNA levels (Figure 6G) were significantly lower in PVN insulin–knockdown mice than in control mice. Taken together, these data suggest that insulin knockdown in the PVN retards the growth of young mice by suppressing pituitary GH production.

### Chronic RS–induced suppression of PVN insulin expression triggers growth retardation in young mice by attenuating pituitary GH production.

**Figure 7:**
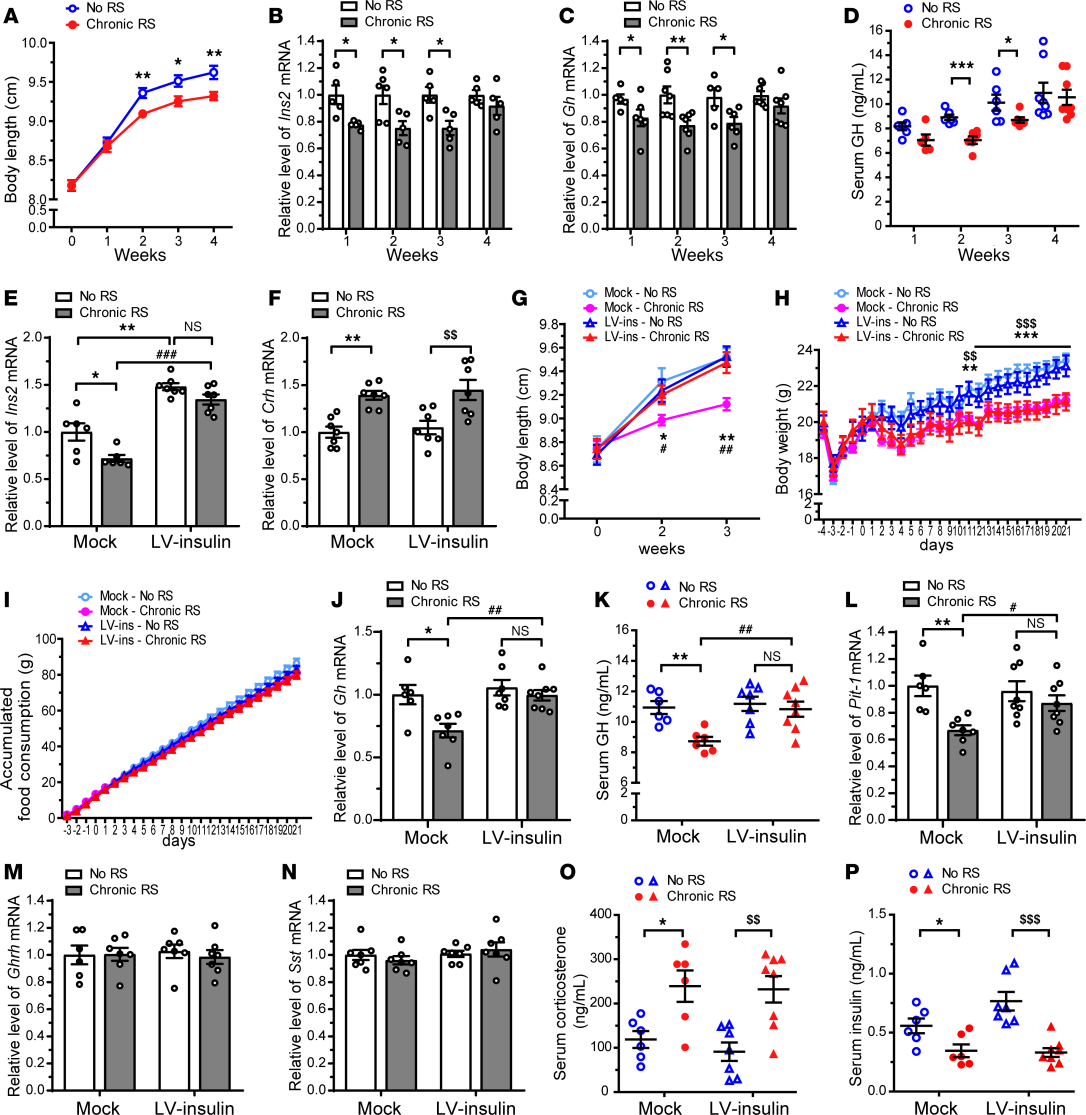
Insulin overexpression in the PVN of young mice prevents chronic restraint stress–induced growth retardation. (**A**–**D**) Young mice were subjected to chronic RS (2 h/d for 1, 2, 3, or 4 weeks). Body length from nose to rump (**A**). Relative hypothalamic *Ins2* (**B**) and pituitary *Gh* (**C**) mRNA levels. Serum GH concentrations (**D**). Data are shown as mean ± SEM, 2-tailed unpaired Student’s *t* test. *P < 0.05, **P < 0.01, ***P < 0.001, *n* = 6–8 mice/group. (**E**–**P**) Lentiviral vector expressing GFP (empty vector, mock) or GFP and *Ins2* (LV-insulin) was injected into the PVN of young mice. Four days after the injection, mice were exposed to chronic RS (2 h/d for 3 weeks). Relative hypothalamic *Ins2* (**E**) and *Crh* (**F**) mRNA levels. Body length from nose to rump (**G**). Body weight (**H**). Accumulated food consumption (**I**). Relative pituitary *Gh* mRNA levels (**J**). Serum GH concentrations (**K**). Relative pituitary *Pit-1* mRNA levels (**L**). Relative hypothalamic *Ghrh* (**M**) and *Sst* (**N**) mRNA levels. Serum corticosterone (**O**) and insulin (**P**) concentrations. Data are shown as mean ± SEM, 2-way ANOVA with Bonferroni’s post hoc tests. * and ^#^*P* < 0.05; **, ^##^, and ^$$^*P* < 0.01; ***, ^###^, and ^$$$^*P* < 0.001, *n* = 5–8 mice/group. *, **, and *** indicate significance of the differences between mock mice under no stress (mock - No RS) and mock mice exposed to chronic RS (mock - Chronic RS). ^#^, ^##^, and ^###^ indicate significance of the differences between mock - Chronic RS and LV-insulin - Chronic RS mice. ^$$^ and ^$$$^ indicate significance of the differences between LV-insulin - No RS and LV-insulin - Chronic RS mice.

We next tested the hypothesis that stress would impede growth of young mice by suppressing pituitary GH production via decreased PVN insulin levels. We subjected young mice to chronic RS (2 h/d for 1, 2, 3, or 4 weeks) and observed a significant inhibition of the gain in body length (Figure 7A). Hypothalamic *Ins2* mRNA expression was significantly lower at 1, 2, and 3 weeks in chronic-RS than in control mice (Figure 7B). In line with decreased hypothalamic *Ins2* mRNA expression, chronic RS reduced or tended to reduce both pituitary *Gh* mRNA and serum GH levels (Figure 7, C and D).

To determine whether chronic RS–induced growth retardation can be attributed to decreased PVN insulin expression, young mice were injected with *Ins2*-overexpressing lentiviruses into the PVN, followed by exposure to chronic RS (2 h/d for 3 weeks). Chronic RS–induced reduction in hypothalamic *Ins2* mRNA expression was blocked (Figure 7E), and of note, the growth retardation was prevented (Figure 7G) by PVN insulin overexpression. Chronic RS upregulated hypothalamic *Crh* mRNA expression (Figure 7F) and significantly slowed down weight gain (Figure 7H) in both mock and LV-insulin mice. Food consumption was similar between mock and LV-insulin mice regardless of chronic RS (Figure 7I). The chronic RS–induced decrease in pituitary *Gh* mRNA and serum GH levels was abolished by insulin overexpression in the PVN (Figure 7, J and K). Furthermore, *Pit-1* mRNA levels were not reduced by chronic RS in LV-insulin as opposed to mock mice (Figure 7L). On the other hand, hypothalamic *Ghrh* and *Sst* mRNA levels were unaffected by insulin overexpression in the PVN under either control or chronic-RS conditions (Figure 7, M and N). Serum corticosterone levels were elevated (Figure 7O), whereas serum insulin levels were substantially diminished (Figure 7P) by chronic RS in both mock and LV-insulin mice. Taken together, these findings suggest that chronic RS stunts the growth of young mice by inhibiting pituitary GH production via the downregulation of insulin expression in the PVN, whereas overexpression of insulin in the PVN prevents chronic RS–induced growth retardation.

## Discussion

In this study, we demonstrated for the first time to our knowledge that in the mouse hypothalamus, insulin-expressing neurons are present in the PVN and transport insulin through axonal projections to the ME. Furthermore, we found that in both adult and young mice, normal PVN insulin expression is important in maintaining pituitary GH gene expression and secretion. Of note, RS-induced inhibition of PVN insulin expression decreases both the pituitary *Gh* mRNA and serum GH levels. Physical or psychological stress is well known to cause growth retardation in children (52–55). However, the specific mechanism underlying this growth retardation has not been clearly elucidated. Our findings suggest that decreased PVN insulin levels under stress conditions underlie growth retardation in young mice by suppressing pituitary GH production. Thus, our study helps further understanding of stress-induced growth retardation that might happen during childhood.

Then, how does PVN insulin regulate pituitary GH production? Peripheral insulin has been considered a negative regulator of pituitary GH gene expression and secretion because pituitary GH production is suppressed by the exposure of a pituitary cell line to insulin in vitro, peripheral injection of insulin into animals, and hyperinsulinemia in obese animals (56–59). High concentrations of insulin inhibit GH production via an increase in SST secretion and a decrease in GHRH secretion from the hypothalamus (60). In contrast, insulin-induced hypoglycemia is known to be a potent stimulus for GH release in humans (61, 62). However, there is an emerging view that insulin may be required to maintain the level and sensitivity of GHRH receptor, thus regulating the action of GHRH on GH-secreting cells and promoting the release of GH from pituitary under normal physiological conditions (60). Our results are in line with this new notion and suggest that PVN insulin is necessary to maintain normal GH level. Furthermore, immunoblot analysis of p-Akt suggests that PVN insulin may regulate pituitary GH production by modulating insulin signaling independently of peripheral insulin.

GHRH, SST, and glucocorticoids, which are major GH regulators, control GH production by acting directly on GH-secreting cells of the anterior pituitary (45–48). However, our results demonstrate that the reduction in pituitary GH production by PVN insulin knockdown is not due to alterations in GHRH, SST, or glucocorticoid levels, since PVN insulin knockdown did not change hypothalamic *Ghrh* and *Sst* mRNA contents or serum corticosterone concentrations. Similarly, our results suggest that in mice overexpressing insulin in the PVN, the blockade of RS-induced reduction in pituitary GH production is not mediated by GHRH, SST, or glucocorticoids. These findings imply the possibility that PVN insulin may also act directly on GH-secreting cells in the anterior pituitary to regulate GH production.

Although proinsulin was expressed predominantly in the neuronal somata in the PVN, proinsulin immunoreactivity was also observed in non-neuronal cells in the hypothalamus. Proinsulin immunoreactivity was detected in ependymal cells lining the 3V. This finding is in accordance with previous reports that ependymal cells produce insulin (63, 64). Proinsulin immunoreactivity was found in all amoeboid microglial cells in the PVN, although there were very few of these cells. Microglial cell activation is reflected in their morphological change from ramified to amoeboid (65). Thus, this observation suggests that insulin could also be produced by activated microglia in the PVN. Further experiments will be needed to determine the functions of insulin derived from non-neuronal cells in the hypothalamus.

Another interesting point that warrants further study is the potential interaction of PVN insulin and the hypothalamic-pituitary-adrenal (HPA) axis. The HPA axis plays a critical role in mediating stress responses, and the PVN is the starting point of this axis regulation (22, 23). In response to stress, PVN CRH neurons are activated and increase the synthesis and secretion of CRH, leading to elevation of serum glucocorticoid levels. In this study, we observed coexpression and cotransportation of insulin and CRH in a significant population of the PVN neurons. However, insulin and CRH genes show a reciprocal expression pattern in the PVNs of mice subjected to acute RS, and modulation of PVN insulin does not affect serum corticosterone levels, suggesting that PVN insulin expression does not correlate with HPA axis activity in stressful situations. Further study will be needed to confirm this conjecture.

In summary, PVN insulin neurons send their neurosecretory nerve terminals to the external zone of the ME, transporting insulin to the anterior pituitary, where it stimulates GH gene expression and secretion, contributing to the increase in body length. Given that PVN insulin overexpression prevented chronic RS–induced growth retardation, our study proposes the role of PVN insulin as a positive regulator of pituitary GH production.

## Methods

### Animal procedures

#### Animals.

Male C57BL/6 mice (3 or 7 weeks old) were purchased from Koatech. Mice were housed under a 12-hour light/12-hour dark cycle (lights on from 7:00 am to 7:00 pm) in individually ventilated cages (1 mouse per cage) with chip bedding at 23°C ± 3°C and a relative humidity of 50% ± 10%. Sterilized food and water were provided ad libitum. Changes in food intake were monitored individually. Adult mice (8–12 weeks old) were used in all experiments except growth observation studies. *Ins1*^+/+^ *Ins2*^+/−^ and *Ins1*^+/+^ *Ins2*^−/−^ mice were originally generated by the group of Jacques Jami (INSERM, France) and were bred and obtained from the University of British Columbia (66, 67). Because *Ins2* was disrupted by inserting the *LacZ* gene at the *Ins2* locus under the control of the *Ins2* promoters, these mice had β-gal knocked into their endogenous *Ins2* locus.

#### Injection (i.p.) of FG.

FG is a nonviral, fluorescent retrograde axonal tracer. The ME is located below the 3V, and to target the ME, the needle must pass through the 3V during the stereotaxic injection. Therefore, in this process, FG may spread to other unintended brain regions through the 3V. Because FG circulating in the blood is retrogradely transported to neurons that send their axon terminals to the external zone of the ME, IP injection of FG has been conventionally used in many studies as an experimental method to see whether a specific hypothalamic neuronal population projects into the ME (28–30). To label neurons that project to the external zone of the ME, mice were injected i.p. with FG (15 μg/g body weight in 100 μL 0.9% saline). Five days after the injection, mice were perfused, and their brains were processed for double immunostaining for proinsulin and FG as described below.

#### Injection of GFP-expressing lentivirus into the PVN.

For anterograde axonal tracing, GFP-expressing lentivirus was bilaterally injected into the PVN using a Hamilton syringe under deep anesthesia with Zoletil 50 (Virbac) and Rompun (Bayer Korea). The stereotaxic coordinates for the PVN were 0.82 mm posterior to bregma, 0.21 mm lateral to the midline, and 5.30 mm below the surface of the skull. Two weeks later, mice were perfused, and their brains were processed for C-peptide immunostaining as described below.

#### Injection (i.c.v.) of colchicine.

Colchicine blocks rapid axonal transport, which results in somatic accumulation of neuropeptides. Colchicine (20 μg; MilliporeSigma) dissolved in 2 μL of 0.01 M PBS was stereotaxically injected into the left lateral ventricle using a Hamilton syringe under deep Zoletil 50/Rompun anesthesia. The stereotaxic coordinates for the lateral ventricle were 0.58 mm posterior to bregma, 1.5 mm lateral to the midline, and 2.5 mm ventral to the skull surface. After 48 hours, vehicle- or colchicine-treated mice were perfused, and their brains were processed for double immunostaining of proinsulin and C-peptide or mCRH and C-peptide as described below. The dose and exposure time of colchicine used in this experiment were based on the previous axonal transport studies with mice (30, 68, 69).

#### Food deprivation.

Fasting reduces enzymatic activities of PC1 and PC2 in the PVN (31). To inhibit these activities, mice were deprived of food for 24 hours from 7:00 pm but were allowed free access to water. Control mice were given ad libitum access to food and water. Fed and fasted mice were sacrificed to collect microdissected PVNs or were perfused and their brains processed for proinsulin and C-peptide immunostaining as described below.

#### Restraint stress.

Mice were subjected to acute RS by physical immobilization in a well-ventilated, 50 mL, conical plastic tube for 15 minutes, 30 minutes, 1 hour, 2 hours, 4 hours, or 8 hours (RS group). Control mice were allowed to roam freely in their home cage but were deprived of food and water (no RS). Mice in both groups were sacrificed to collect the hypothalamus and pituitary for mRNA expression analysis or were perfused and their brains processed for triple immunostaining of proinsulin, CRH, and c-Fos or double immunostaining of C-peptide and CRH as described below. For exposure to chronic RS, young mice (4 weeks old) were immobilized as above for 2 hours daily (11:00 am to 1:00 pm) for 1, 2, 3, or 4 weeks, and body length from nose to rump was measured every week. Following the last measurement, mice were sacrificed to collect blood, hypothalamus, and pituitary.

### Immunofluorescence staining

Mice were deeply anesthetized with an i.p. injection of a cocktail containing Zoletil 50 and Rompun, then perfused transcardially with PBS (pH 7.4) at 37°C, followed by 4% paraformaldehyde in PBS (pH 7.4; shifted from 37°C [15 mL] to ice cold [10 mL]). Brain and pancreas were quickly dissected out, postfixed with the same fixative for 4 hours at 4°C, and immersed in 30% sucrose in PBS (pH 7.4) until they sank. To prepare frozen sections, the brain and pancreas were embedded in optimal cutting temperature compound (CellPath), frozen on dry ice. Serial coronal brain sections (30–40 μm) and pancreas sections (12 μm) were cut on a cryostat (Thermo Fisher Scientific) and extensively rinsed in PBS.

The sections were incubated in combinations of primary antibodies diluted in PBS with 5% donkey serum and 0.3% Triton X-100 for 16–72 hours at 4°C. The final dilutions of primary antibodies were as follows: mouse anti-proinsulin (1:50; R&D Systems, Bio-Techne, MAB13361; recognizes proinsulin but not mature insulin or C-peptide), guinea pig anti-insulin (1:100; Abcam, ab7842; recognizes both proinsulin and mature insulin), rabbit anti–C-peptide (1:300; Cell Signaling Technology, 4593; recognizes C-peptide but not proinsulin or mature insulin), rabbit anti–β-gal (1:100; Thermo Fisher Scientific, A-11132), rabbit anti-NeuN (1:200; Cell Signaling Technology, 24307), chicken anti-MAP2 (1:1000; Abcam, ab5392), rabbit anti-GFAP (1:800; Dako, Z0334), mouse anti-GFAP (1:800; MilliporeSigma, MAB360), goat anti–Iba-1 (1:500; Abcam, ab5076), guinea pig anti-synapsin (1:500; Synaptic Systems, 106 004), chicken anti-vimentin (1:2500; MilliporeSigma, AB5733), rabbit anti-FG (1:10,000; Fluorochrome), goat anti-mCRH (1:200; Santa Cruz Biotechnology, Inc., sc-1759; recognizes only the mature form), guinea pig anti-CRH (1:600; Peninsula Laboratories International, Inc., T-5007; recognizes both the precursor and mature form), goat anti-SST (1:200; Santa Cruz Biotechnology, Inc., sc-7819), rabbit anti–c-Fos (1:1000; Santa Cruz Biotechnology, Inc., sc-52), and rabbit anti-GFP (1:1000; Abcam, ab290).

After extensive rinsing in PBS with 0.01% Tween-20, fluorophore-conjugated secondary antibodies (1:200–500; Jackson ImmunoResearch, 715-545-150, 706-545-148, 711-165-152, 715-165-150, 705-165-147, 703-545-155, 706-165-148, 705-545-147, 706-475-148, and 711-545-152) were applied for 1–2 hours at room temperature (RT). The sections were subsequently incubated for 5 minutes with 1 μg/mL Hoechst 33258 (Invitrogen, Thermo Fisher Scientific) in PBS for nuclear staining, mounted on glass slides, and coverslipped with VECTASHIELD Mounting Medium (Vector Laboratories).

Sections were analyzed using an LSM 780 or LSM 800 confocal laser-scanning microscope (Carl Zeiss) with maximal signal separation. To analyze the fluorescence intensity of proinsulin or CRH in the PVN, we used both sides of each of the 3–5 PVN sections per mouse. To analyze the fluorescence intensity of C-peptide or CRH in the ME, we used 3–5 sections from the ME per mouse. Fluorescence intensity was quantified using ImageJ software (NIH) and represented as a ratio of immunoreactivity to background fluorescence. To analyze the percentage of proinsulin-positive cells that coexpress CRH or SST, the number of such cells was blindly counted on both sides of each of the 3–5 PVN sections per mouse. To analyze the percentage of CRH-positive neurons that coexpressed proinsulin, the number of CRH-positive cells with proinsulin was blindly counted on both sides of each of the 3–5 PVN sections per mouse. To analyze the percentage of PVN insulin/CRH-expressing neurons that coexpress c-Fos, the number of proinsulin/CRH co-positive cells with c-Fos was blindly counted on both sides of each of the 3–5 PVN sections per mouse.

### In situ hybridization

Brains from transcardially perfused mice were embedded in paraffin as described previously with some modifications (70). Serial brain sections (5 μm) were obtained from the paraffin-embedded brain blocks. The sections were then deparaffinized with xylene, boiled in pretreatment solution for 10 minutes, and digested with Protease QF (Affymetrix) for 10 minutes at 40°C. To determine localization of *Ins2* mRNA in the hypothalamus, the ViewRNA ISH tissue assay kit (Affymetrix) was used as recommended by the manufacturer. The sections were hybridized with mouse antisense *Ins2* (Affymetrix, VB1-10063) and sense *Ins2* (Affymetrix, VB1-20038) Fast Red probes for 4 hours at 40°C. Slides were then incubated with Label Probe 1-AP Type 1 solution and Fast Red substrate. Coverslips were mounted on slides, and images were acquired using an ECLIPSE 90i light microscope (Nikon).

### Electron microscopic immunohistochemistry

Ultrathin ME sections were collected on 300-mesh nickel grids as described previously (71). Grids were etched for 5 seconds in sodium ethanolate (a saturated solution of NaOH in 100% ethanol) at least 24 hours before use. Grids were rinsed in TBS with 0.1% Triton X-100 (TBST; pH 7.4), then incubated for 20 minutes in 2% human serum albumin (MilliporeSigma) containing 0.1% sodium borohydride and 50 mM glycine (pH 7.4). Grids were then washed in TBST and incubated for 3 hours at RT in a mixture of goat anti-mCRH (1:80; Santa Cruz Biotechnology) and rabbit anti–C-peptide (1:30; Cell Signaling Technology) antibodies. Grids were rinsed in TBST, incubated with 12 nm gold–conjugated donkey anti–goat IgG antibody (1:25 in TBST containing 0.05% polyethylene glycol; Jackson ImmunoResearch, 705-205-147) for 2 hours, rinsed in TBST, and incubated with 30 nm gold–conjugated goat anti–rabbit IgG antibody (1:25 in TBST containing 0.05% polyethylene glycol; BBI Solutions, EM.GAR30) for 2 hours. After rinsing in distilled water, the grids were stained with uranyl acetate and lead citrate and examined on a Hitachi H-7500 electron microscope at 80 kV accelerating voltage. Images were captured with Digital Montage software driving a MultiScan cooled ES1000W Erlangshen CCD Camera (Gatan, Inc.) attached to the microscope.

### Preparation of lentiviral shRNAs and *Ins2*-overexpressing lentiviral vector

GIPZ nonsilencing lentiviral shRNA control (Dharmacon, RHS4346), 4 different mouse *Ins2* shRNAs (Dharmacon, V3LMM 471259, 471262, 471263, and 471364) that were cloned into the GIPZ lentiviral vector, and an *Ins2*-overexpressing lentiviral vector (GeneCopoeia, EX-Mn03325-LV205) were used for gene delivery. The pMD2.G and psPAX2 vectors were used as an envelope and packaging vector, respectively, to produce lentivirus in the Lenti-X 293T cell line (Clontech Laboratories). Transient transfections were performed with the TurboFect transfection reagent (Thermo Fisher Scientific) following the manufacturer’s protocol. Lentivirus-containing cell supernatants were collected 72 hours posttransfection, filtered through 0.45 μm syringe filters, loaded onto 20% sucrose solution, and concentrated by ultracentrifugation at 40,000 *g* for 90 minutes at 4°C (72). Viral pellets were suspended in PBS. Lentivirus was titrated by counting GFP-positive cells on an Accuri C6 flow cytometer (BD Biosciences). Lentiviral vectors were diluted to 0.3 × 10^10^ to 1 × 10^10^ transducing units/mL.

### Administration of lentiviral shRNAs or *Ins2*-overexpressing lentivirus into the PVN

Lentiviral vectors for knockdown or overexpression of *Ins2* (1 μL each) were administered into the PVN of adult mice (8 weeks old). For adult mice, the stereotaxic coordinates for bilateral injections were as described above for GFP-expressing lentivirus injections. The coordinates for young mice were adjusted to 0.74 mm caudal to bregma, 0.20 mm lateral to the midline, and 4.75 mm below the surface of the skull.

After lentivirus administration into the PVN of adult mice, body weight and food consumption were measured in the middle of the light cycle every day. Two weeks after injection, mice were sacrificed to collect blood, pituitary samples, and brains; mice overexpressing insulin in the PVN were subjected to RS for 8 hours before sacrifice as described above.

In the case of young mice, body weight and food consumption were measured in the middle of the light cycle every 2 days for 6 weeks (PVN insulin–knockdown mice) or every day for 3 weeks (mice overexpressing insulin in the PVN). The latter mice were subjected to chronic RS for 3 weeks as described above, starting from 5 days after lentiviral injection. Body length was measured from nose to rump immediately before lentiviral injection and 6 weeks after the injection (PVN insulin–knockdown mice) or every week (mice overexpressing insulin in the PVN). Following the last measurement of body length, mice were sacrificed to collect blood, the hypothalamus, and the pituitary.

### Microdissection of PVN for RNA extraction

Brains were rapidly dissected out, embedded in optimal cutting temperature compound, and frozen on dry ice. Frozen sections (120 μm) were obtained on a cryostat. PVN-containing serial coronal brain sections between bregma levels –0.58 and –0.94 mm were placed on cover glasses. The PVN regions were then microdissected bilaterally using a 0.5 mm–diameter brain punch (Stoelting Co.). Microdissected PVN samples were expelled into microcentrifuge tubes and frozen in liquid nitrogen. PVNs of 3 mice were pooled to extract RNA.

### RNA extraction and gene expression analysis by qRT-PCR

TRIzol reagent (Ambion, Thermo Fisher Scientific) and chloroform were used to extract total RNA from each tissue. Total hypothalamic tissues were used for analysis of *Ins2*, *Crh*, *Ghrh*, and *Sst* mRNA. Microdissected PVN samples were used for analysis of *Ins2* and *Crh* mRNA. Total pituitary gland tissues were used for analysis of proopiomelanocortin (*Pomc*), prolactin, follicle-stimulating hormone β (*FshB*), luteinizing hormone β (*LhB*), *Gh*, and *Pit-1* mRNA. Using 1 μg of total RNA extracted from each sample, cDNA was synthesized as described previously (73). cDNA from total hypothalamic and total pituitary tissues was diluted 1:5 and 1:20 in nuclease-free water, respectively. Two microliters of diluted cDNA was used as a template for qRT-PCR, which was performed according to the SYBR Green protocol (Takara Bio) in a CFX-96 qRT-PCR machine (Bio-Rad Laboratories). Sequences of primers used in qRT-PCR are listed in Table 1. The ΔΔCt method was used to determine the relative expression level of each target gene after normalization to that of the *Gapdh* gene. The Ct values for *Ins2* mRNA were 29.779 ± 0.122 in total hypothalamic tissues and 30.735 ± 0.143 in microdissected PVN samples under 40 thermal cycles.

### Serum GH, corticosterone, and insulin analysis

Serum GH, corticosterone, and insulin levels were measured by ELISA. Blood samples were collected by tail bleeding at 0500, 1100, 1700, and 2300 hours or heart puncture at 1700 hours, clotted for 1 hour at RT, and centrifuged for 20 minutes at 2000 *g* to separate serum from blood clots. Blood samples collected by tail bleeding were used for GH ELISA, and blood samples collected by heart puncture were used for GH, corticosterone, and insulin ELISA. A mouse GH ELISA kit (LifeSpan Biosciences), a mouse corticosterone ELISA kit (ALPCO Diagnostics), or a mouse insulin ELISA kit (ALPCO Diagnostics) was used, respectively, following the manufacturers’ protocols.

### Immunoblot analysis

To extract protein from total pituitary tissues, samples were dissolved in lysis buffers as described previously (68). Lysates were separated on 10% SDS-PAGE and blotted onto polyvinylidene difluoride membranes (MilliporeSigma, IPVH00010) for 40 minutes at 20 V in transfer buffer containing 25 mM Tris base, 192 mM glycine, and 10% methanol. The membranes were blocked with 5% skim milk for 1 hour and then incubated with primary antibodies against p-Akt (1:1000; Cell Signaling Technology, 4060), Akt (1:1000; Cell Signaling Technology, 9272), or GAPDH (1:10,000; Cell Signaling Technology, 2118) at 4°C overnight. After extensive washing in Tris-buffered saline with 0.1% Tween-20, the membranes were incubated with anti-rabbit horseradish peroxidase secondary antibody (Thermo Fisher Scientific, NCI1460KR) and visualized by ECL solutions (Thermo Fisher Scientific, NCI4080KR) according to the manufacturer’s procedure. Band intensities were quantified using ImageJ software (NIH).

For immunoblot assay of proinsulin, mice were perfused with PBS only before collecting the samples. The pancreatic protein was extracted from 1 WT and *Ins1*^+/+^ *Ins2*^−/−^ mouse, respectively. Also, in both WT and *Ins1*^+/+^ *Ins2*^−/−^ mice, the total hypothalamus of 3 mice and the microdissected PVNs of 7 mice were pooled and concentrated to extract protein, respectively. Lysates were separated on 15% SDS-PAGE and blotted onto polyvinylidene difluoride membranes for 30 minutes at 16 V in the transfer buffer. The membranes were blocked and then incubated with primary antibody against proinsulin (1:1000; Cell Signaling Technology, 8138) at 4°C overnight. After extensive washing, the membranes were incubated with anti-mouse horseradish peroxidase secondary antibody (Jackson ImmunoResearch, 115-035-003) and visualized by the ECL solutions.

### Experimental design

In situ hybridization and immunostaining analyses were performed with 3–5 PVN or ME sections from each mouse (at least 3 mice), and representative data were presented. For each experimental condition, we calculated the sample size from the results of pilot experiments with 3 mice per group using G*Power 3.1.9.2 (74). Considering the possible loss of mice during experiments, the number of mice per experiment was greater than but less than twice the calculated sample size, with one exception: we used 15 mice to obtain enough PVN samples for microdissection, although the calculated sample size was 4. Final sample sizes (*n*) are indicated in the legend of each figure.

### Statistics

Statistical analyses were carried out using GraphPad Prism 8 software. Statistical comparisons between a control group and an experimental group were made using 2-tailed unpaired Student’s *t* test. Multiple comparisons in time course study of hypothalamic *Crh* and *Ins2* mRNA expression were determined using 1-way ANOVA followed by Bonferroni’s post hoc tests. Multiple comparisons in PVN insulin overexpression data were made using 2-way ANOVA followed by Bonferroni’s post hoc tests. Data are presented as mean ± SEM and significance was determined at *P* < 0.05.

### Study approval

All animal experiments were conducted in accordance with the guidelines on animal care and use as approved by the Institutional Animal Care and Use Committee of Daegu Gyeongbuk Institute of Science and Technology. For electron microscopic immunohistochemistry, animal procedures were performed according to the NIH guidelines and were approved by the Kyungpook National University Intramural Animal Care and Use Committee. For immunofluorescence staining using *Ins1*^+/+^ *Ins2*^+/−^ and *Ins1*^+/+^ *Ins2*^−/−^ mice, animal protocols were approved by the University of British Columbia Animal Care Committee in accordance with national and international guidelines.

## Author contributions

EKK, JL, and KK conceived and designed the experiments. JL, KK, and JYB performed the experiments. EKK, JL, KK, JHC, and YCB analyzed and interpreted the data. TPO and JDJ provided the tissues of *Ins1*^+/+^ *Ins2*^+/−^ and *Ins1*^+/+^ *Ins2*^−/−^ mice, helped design some experiments and interpret data, and edited the manuscript. EKK and KK wrote the manuscript with input from all authors.

## Supplementary Material

Supplemental data

## Figures and Tables

**Table 1:**
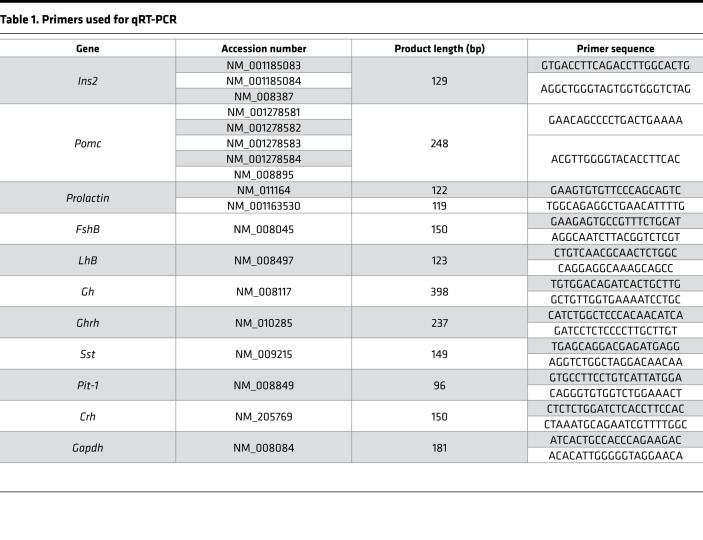
Primers used for qRT-PCR

## Version 1 07/09/2020

In-Press Preview

## Version 2 08/20/2020

Electronic publication

## References

[1] Banks WA, Owen JB, Erickson MA (2012). Insulin in the brain: there and back again. Pharmacol Ther.

[2] Grunblatt E, Salkovic-Petrisic M, Osmanovic J, Riederer P, Hoyer S (2007). Brain insulin system dysfunction in streptozotocin intracerebroventricularly treated rats generates hyperphosphorylated tau protein. J Neurochem.

[3] Havrankova J, Schmechel D, Roth J, Brownstein M (1978). Identification of insulin in rat brain. Proc Natl Acad Sci U S A.

[4] Young WS (1986). Periventricular hypothalamic cells in the rat brain contain insulin mRNA. Neuropeptides.

[5] Mehran AE (2012). Hyperinsulinemia drives diet-induced obesity independently of brain insulin production. Cell Metab.

[6] Mazucanti CH (2019). Release of insulin produced by the choroid plexis is regulated by serotonergic signaling. JCI Insight.

[7] Molnar G (2014). GABAergic neurogliaform cells represent local sources of insulin in the cerebral cortex. J Neurosci.

[8] Kuwabara T (2011). Insulin biosynthesis in neuronal progenitors derived from adult hippocampus and the olfactory bulb. EMBO Mol Med.

[9] Nemoto T (2014). New insights concerning insulin synthesis and its secretion in rat hippocampus and cerebral cortex: amyloid-β1-42-induced reduction of proinsulin level via glycogen synthase kinase-3β. Cell Signal.

[10] Devaskar SU, Giddings SJ, Rajakumar PA, Carnaghi LR, Menon RK, Zahm DS (1994). Insulin gene expression and insulin synthesis in mammalian neuronal cells. J Biol Chem.

[11] Devaskar SU, Singh BS, Carnaghi LR, Rajakumar PA, Giddings SJ (1993). Insulin II gene expression in rat central nervous system. Regul Pept.

[12] Deltour L (1993). Differential expression of the two nonallelic proinsulin genes in the developing mouse embryo. Proc Natl Acad Sci U S A.

[13] Li L (2016). Knockin of Cre Gene at Ins2 locus reveals no cre activity in mouse hypothalamic neurons. Sci Rep.

[14] Madadi G, Dalvi PS, Belsham DD (2008). Regulation of brain insulin mRNA by glucose and glucagon-like peptide 1. Biochem Biophys Res Commun.

[15] Lee J, Kim K, Yu SW, Kim EK (2016). Wnt3a upregulates brain-derived insulin by increasing NeuroD1 via Wnt/β-catenin signaling in the hypothalamus. Mol Brain.

[16] Eigler T, Ben-Shlomo A (2014). Somatostatin system: molecular mechanisms regulating anterior pituitary hormones. J Mol Endocrinol.

[17] Kawano H, Daikoku S, Shibasaki T (1988). CRF-containing neuron systems in the rat hypothalamus: retrograde tracing and immunohistochemical studies. J Comp Neurol.

[18] Merchenthaler I, Liposits Z (1994). Mapping of thyrotropin-releasing hormone (TRH) neuronal systems of rat forebrain projecting to the median eminence and the OVLT. Immunocytochemistry combined with retrograde labeling at the light and electron microscopic levels. Acta Biol Hung.

[19] Lennard DE, Eckert WA, Merchenthaler I (1993). Corticotropin-releasing hormone neurons in the paraventricular nucleus project to the external zone of the median eminence: a study combining retrograde labeling with immunocytochemistry. J Neuroendocrinol.

[20] Ghamari-Langroudi M, Vella KR, Srisai D, Sugrue ML, Hollenberg AN, Cone RD (2010). Regulation of thyrotropin-releasing hormone-expressing neurons in paraventricular nucleus of the hypothalamus by signals of adiposity. Mol Endocrinol.

[21] Horn AM, Robinson IC, Fink G (1985). Oxytocin and vasopressin in rat hypophysial portal blood: experimental studies in normal and Brattleboro rats. J Endocrinol.

[22] Guilliams TG, Edwards L (2010). Chronic stress and the HPA axis. The Standard.

[23] Jankord R, Herman JP (2008). Limbic regulation of hypothalamo-pituitary-adrenocortical function during acute and chronic stress. Ann N Y Acad Sci.

[24] Kobayashi H, Matsui T, Ishii S (1970). Functional electron microscopy of the hypothalamic median eminence. Int Rev Cytol.

[25] Buma P, Nieuwenhuys R (1988). Ultrastructural characterization of exocytotic release sites in different layers of the median eminence of the rat. Cell Tissue Res.

[26] Swanson LW, Kuypers HG (1980). The paraventricular nucleus of the hypothalamus: cytoarchitectonic subdivisions and organization of projections to the pituitary, dorsal vagal complex, and spinal cord as demonstrated by retrograde fluorescence double-labeling methods. J Comp Neurol.

[27] Armstrong WE, Warach S, Hatton GI, McNeill TH (1980). Subnuclei in the rat hypothalamic paraventricular nucleus: a cytoarchitectural, horseradish peroxidase and immunocytochemical analysis. Neuroscience.

[28] Merchenthaler I (1990). Retrograde labeling of hypophysiotropic neurons by local injection of wheat germ agglutinin (WGA) into the median eminence or peripheral administration of fluoro-gold. Mol Cell Neurosci.

[29] Merchenthaler I (1991). Neurons with access to the general circulation in the central nervous system of the rat: a retrograde tracing study with fluoro-gold. Neuroscience.

[30] Kakizawa K (2016). A novel GABA-mediated corticotropin-releasing hormone secretory mechanism in the median eminence. Sci Adv.

[31] Sanchez VC (2004). Regulation of hypothalamic prohormone convertases 1 and 2 and effects on processing of prothyrotropin-releasing hormone. J Clin Invest.

[32] Gonzalez-Hernandez T (2006). Interleukin-6 and nitric oxide synthase expression in the vasopressin and corticotrophin-releasing factor systems of the rat hypothalamus. J Histochem Cytochem.

[33] Cortes R, Ceccatelli S, Schalling M, Hokfelt T (1990). Differential effects of intracerebroventricular colchicine administration on the expression of mRNAs for neuropeptides and neurotransmitter enzymes, with special emphasis on galanin: an in situ hybridization study. Synapse.

[34] Dong W, Seidel B, Marcinkiewicz M, Chretien M, Seidah NG, Day R (1997). Cellular localization of the prohormone convertases in the hypothalamic paraventricular and supraoptic nuclei: selective regulation of PC1 in corticotrophin-releasing hormone parvocellular neurons mediated by glucocorticoids. J Neurosci.

[35] Weiss M, Steiner DF, Philipson LH. Insulin biosynthesis, secretion, structure, and structure-activity relationships. In: Feingold KR, et al, eds. *Endotext*. MDText.com, Inc.; 2000. 25905258

[36] Uchoa ET, Aguilera G, Herman JP, Fiedler JL, Deak T, de Sousa MB (2014). Novel aspects of glucocorticoid actions. J Neuroendocrinol.

[37] Kalin NH, Takahashi LK, Chen FL (1994). Restraint stress increases corticotropin-releasing hormone mRNA content in the amygdala and paraventricular nucleus. Brain Res.

[38] Flak JN, Ostrander MM, Tasker JG, Herman JP (2009). Chronic stress-induced neurotransmitter plasticity in the PVN. J Comp Neurol.

[39] Makino S, Smith MA, Gold PW (1995). Increased expression of corticotropin-releasing hormone and vasopressin messenger ribonucleic acid (mRNA) in the hypothalamic paraventricular nucleus during repeated stress: association with reduction in glucocorticoid receptor mRNA levels. Endocrinology.

[40] Steyn FJ (2011). Development of a method for the determination of pulsatile growth hormone secretion in mice. Endocrinology.

[41] Gahete MD (2013). Insulin and IGF-I inhibit GH synthesis and release in vitro and in vivo by separate mechanisms. Endocrinology.

[42] Karin M, Theill L, Castrillo JL, McCormick A, Brady H (1990). Cell type specific expression of the growth hormone gene and its control by GHF-1. Nihon Naibunpi Gakkai Zasshi.

[43] Gaiddon C, Tian J, Loeffler JP, Bancroft C (1996). Constitutively active G(S) alpha-subunits stimulate Pit-1 promoter activity via a protein kinase A-mediated pathway acting through deoxyribonucleic acid binding sites both for Pit-1 and for adenosine 3’,5’-monophosphate response element-binding protein. Endocrinology.

[44] Bodner M, Castrillo JL, Theill LE, Deerinck T, Ellisman M, Karin M (1988). The pituitary-specific transcription factor GHF-1 is a homeobox-containing protein. Cell.

[45] Brazeau P (1973). Hypothalamic polypeptide that inhibits the secretion of immunoreactive pituitary growth hormone. Science.

[46] Plotsky PM, Vale W (1985). Patterns of growth hormone-releasing factor and somatostatin secretion into the hypophysial-portal circulation of the rat. Science.

[47] Tannenbaum GS, Painson JC, Lengyel AM, Brazeau P (1989). Paradoxical enhancement of pituitary growth hormone (GH) responsiveness to GH-releasing factor in the face of high somatostatin tone. Endocrinology.

[48] Mazziotti G, Giustina A (2013). Glucocorticoids and the regulation of growth hormone secretion. Nat Rev Endocrinol.

[49] Savage MO (1993). Clinical features and endocrine status in patients with growth hormone insensitivity (Laron syndrome). J Clin Endocrinol Metab.

[50] Zhou Y (1997). A mammalian model for Laron syndrome produced by targeted disruption of the mouse growth hormone receptor/binding protein gene (the Laron mouse). Proc Natl Acad Sci U S A.

[51] Efstratiadis A (1998). Genetics of mouse growth. Int J Dev Biol.

[52] Dobrova-Krol NA, van Ijzendoorn MH, Bakermans-Kranenburg MJ, Cyr C, Juffer F (2008). Physical growth delays and stress dysregulation in stunted and non-stunted Ukrainian institution-reared children. Infant Behav Dev.

[53] Fernald LC, Grantham-McGregor SM (2002). Growth retardation is associated with changes in the stress response system and behavior in school-aged jamaican children. J Nutr.

[54] Fernald LC, Grantham-McGregor SM (1998). Stress response in school-age children who have been growth retarded since early childhood. Am J Clin Nutr.

[55] Savendahl L (2012). The effect of acute and chronic stress on growth. Sci Signal.

[56] Melmed S (1984). Insulin suppresses growth hormone secretion by rat pituitary cells. J Clin Invest.

[57] Luque RM, Kineman RD (2006). Impact of obesity on the growth hormone axis: evidence for a direct inhibitory effect of hyperinsulinemia on pituitary function. Endocrinology.

[58] Lanzi R (1999). Elevated insulin levels contribute to the reduced growth hormone (GH) response to GH-releasing hormone in obese subjects. Metabolism.

[59] Lanzi R (1997). Evidence for an inhibitory effect of physiological levels of insulin on the growth hormone (GH) response to GH-releasing hormone in healthy subjects. J Clin Endocrinol Metab.

[60] Qiu H, Yang JK, Chen C (2017). Influence of insulin on growth hormone secretion, level and growth hormone signalling. Sheng Li Xue Bao.

[61] Shibasaki T (1985). Plasma GH responses to GHRH and insulin-induced hypoglycemia in man. J Clin Endocrinol Metab.

[62] Hanew K, Utsumi A (2002). The role of endogenous GHRH in arginine-, insulin-, clonidine- and l-dopa-induced GH release in normal subjects. Eur J Endocrinol.

[63] Dakic TB (2017). Short-term fasting promotes insulin expression in rat hypothalamus. Eur J Neurosci.

[64] Tarrach CHA, Dodson AAR, Foster CS, Harrold JA, Wilding JPH. Rat third ventricle ependymal cells possess the ability to produce insulin. Abstract presented at: 65th Scientiﬁc Sessions Congress; June 10–14, 2005; San Diego, California, USA. Abstract 1528-P

[65] Kettenmann H (2007). Neuroscience: the brain’s garbage men. Nature.

[66] Duvillie B (1997). Phenotypic alterations in insulin-deficient mutant mice. Proc Natl Acad Sci U S A.

[67] Templeman NM (2017). Reduced circulating insulin enhances insulin sensitivity in old mice and extends lifespan. Cell Rep.

[68] Romanov RA (2015). A secretagogin locus of the mammalian hypothalamus controls stress hormone release. EMBO J.

[69] Ryu KY, Garza JC, Lu XY, Barsh GS, Kopito RR (2008). Hypothalamic neurodegeneration and adult-onset obesity in mice lacking the Ubb polyubiquitin gene. Proc Natl Acad Sci U S A.

[70] Yoo SJ (2017). Differential spatial expression of peripheral olfactory neuron-derived BACE1 induces olfactory impairment by region-specific accumulation of β-amyloid oligomer. Cell Death Dis.

[71] Mah W (2017). A role for the purinergic receptor P2X_3_ in astrocytes in the mechanism of craniofacial neuropathic pain. Sci Rep.

[72] Ichim CV, Wells RA (2011). Generation of high-titer viral preparations by concentration using successive rounds of ultracentrifugation. J Transl Med.

[73] Oh TS, Cho H, Cho JH, Yu SW, Kim EK (2016). Hypothalamic AMPK-induced autophagy increases food intake by regulating NPY and POMC expression. Autophagy.

[74] Faul F, Erdfelder E, Buchner A, Lang AG (2009). Statistical power analyses using G*Power 3.1: tests for correlation and regression analyses. Behav Res Methods.

