# Supplementary material for: Insulin synthesized in the paraventricular nucleus of the hypothalamus regulates pituitary growth hormone production
Source: JCI Insight. 2020 Aug 20;5(16):e135412. doi: 10.1172/jci.insight.135412 (PMC7455129; doi:10.1172/jci.insight.135412)
Supplement: Supplemental data [file jciinsight-5-135412-s070.pdf]

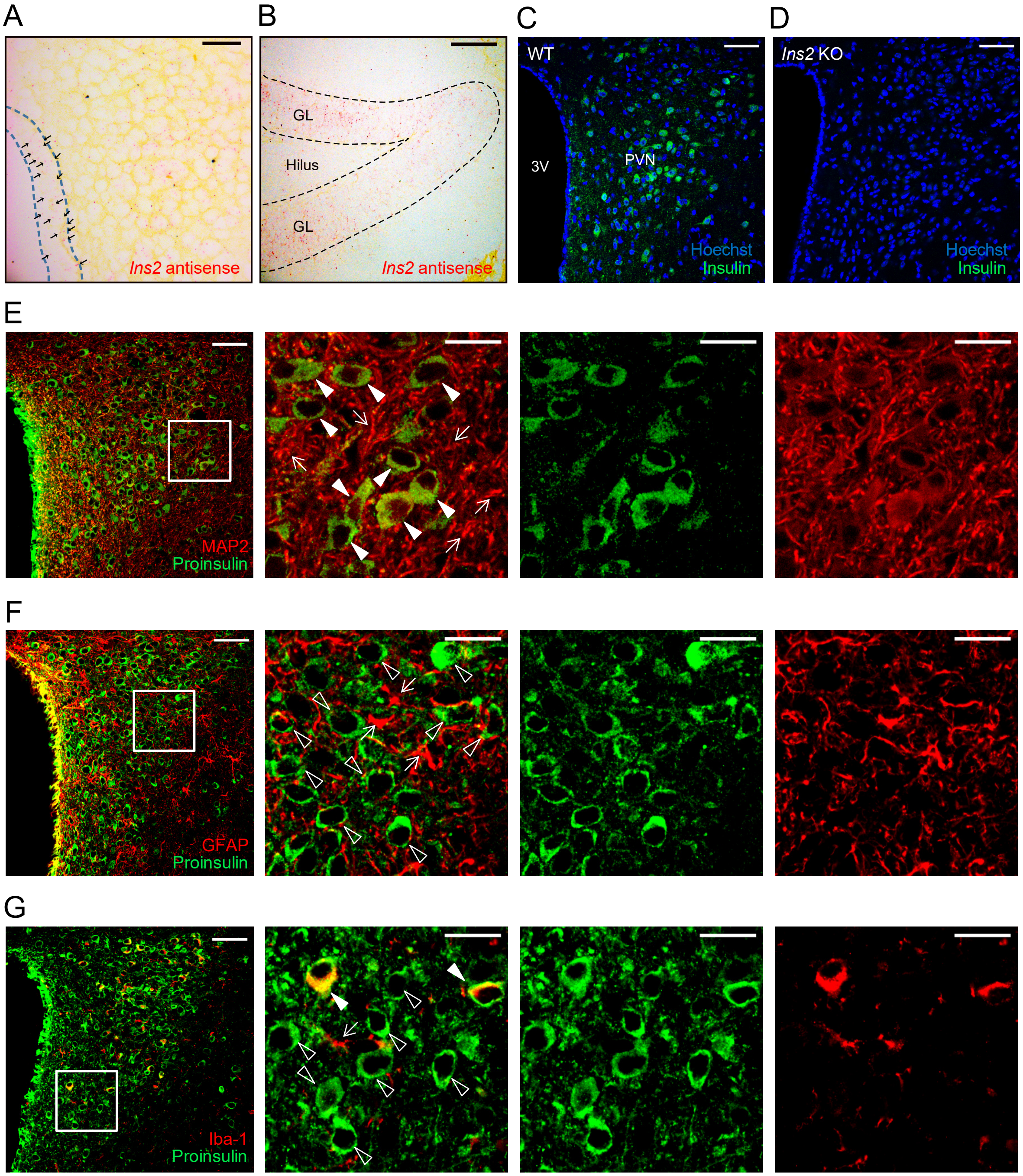
A
B
C
D
Ins2 antisense
GL
Hilus
GL
Ins2 antisense
WT
3V
PVN
Hoechst
Insulin
Ins2 KO
Hoechst
Insulin
E
MAP2
Proinsulin
F
GFAP
Proinsulin
G
Iba-1
Proinsulin

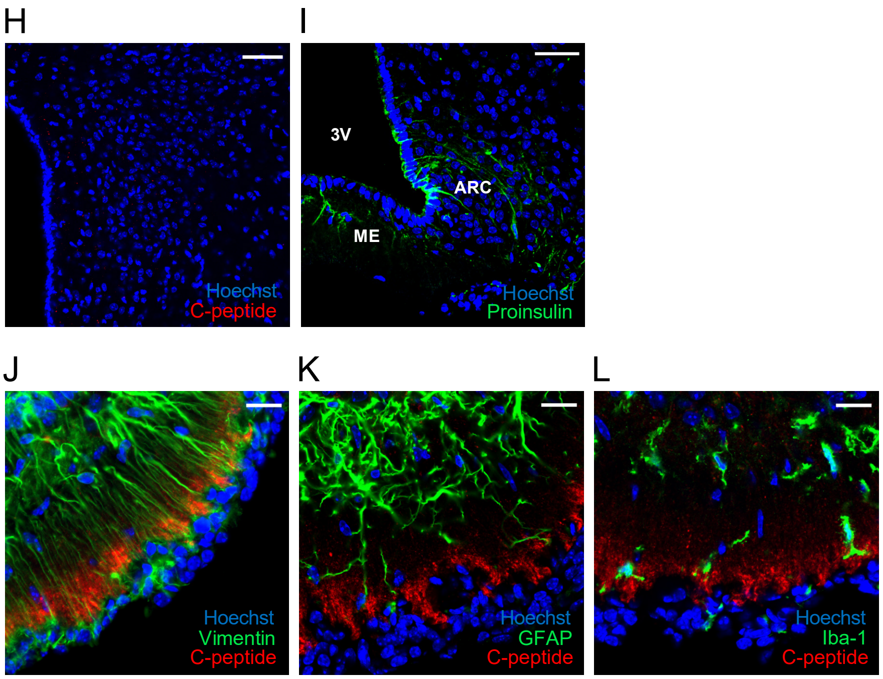

Supplemental Figure 1. Reciprocal expression of proinsulin and C-peptide in the hypothalamus.
(**A**) A representative *in situ* hybridization image showing that *Ins2* mRNA is expressed in ependymal cell layer lining the third ventricle. Dotted lines represent the area of the ependymal cell layer. Arrows indicate *Ins2* mRNA positive signals within the ependymal cell layer. (**B**) A representative hippocampal section hybridized with the *Ins2* antisense probe as a positive control for *in situ* hybridization. The hippocampal granule cell layer showed *Ins2*-positive signals. (**C**) A representative PVN section from WT mice stained by insulin antibody (Abcam, #ab7842; recognizes both proinsulin and mature insulin). (**D**) The insulin antibody failed to stain the PVN from *Ins2* KO mice. (**E-G**) Representative double immunofluorescence images of the PVN. Proinsulin immunoreactivity was found mainly in cell bodies of neurons positive for MAP2 in the PVN (E). Proinsulin was not co-localized with GFAP-positive astrocytes in the PVN (F). Proinsulin was co-localized with Iba-1--positive amoeboid microglia but not with the ramified microglia (G). Arrows indicate single labeling of each cell type marker. Open arrowheads indicate proinsulin single labeling. Solid arrowheads point co-localization of proinsulin and each cell-type marker. (**H**) C-peptide immunostaining in the PVN. (**I**) Proinsulin immunostaining in the external zone of the ME. (**J-L**) Representative double immunofluorescence images of the external zone of the ME. C-peptide was not co--localized with the tanycyte marker vimentin (J), the astrocytes marker GFAP (K), or the microglia marker Iba-1 (L) in the external zone of the ME. Scale bars: 50 μm (B-I), 20 μm (A, J-L, high magnification inset images in E-G).
GL, granule cell layer; 3V, third ventricle; PVN, paraventricular nucleus; Arc, arcuate nucleus; ME, median eminence

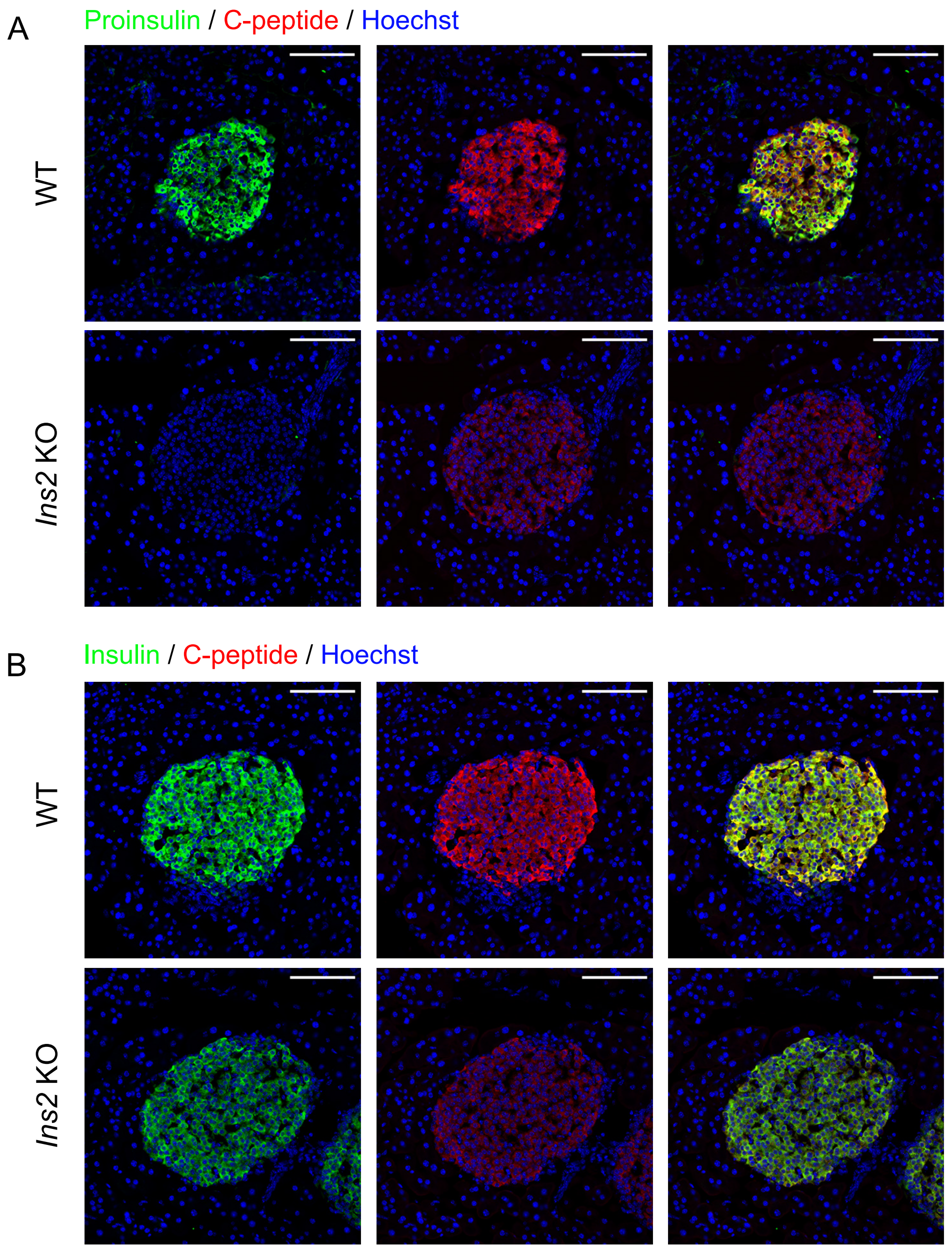

Supplemental Figure 2. Validation of the proinsulin, insulin, and C-peptide antibodies in *Ins2* KO pancreas sections. (**A, B**) Representative pancreas sections from WT and *Ins2* KO mice stained by proinsulin and C-peptide antibodies. The proinsulin antibody (R&D systems, #MAB13361) failed to stain the pancreas of *Ins2* KO mice, suggesting that the anti-body detects only mouse proinsulin 2 not proinsulin 1 form (A). The insulin antibody (Abcam, #ab7842; recognizes both proinsulin and mature insulin) stained the pancreas from *Ins2* KO mice, but the immunoreactivity was reduced slightly (B). C-peptide immunoreactivity detected by the C-peptide antibody (Cell Signaling Technology, #4593) was dramatically diminished in the pancreas of *Ins2* KO mice compared with that of WT mice, but not abolished (A, B).

Scale bars: 100 µm.

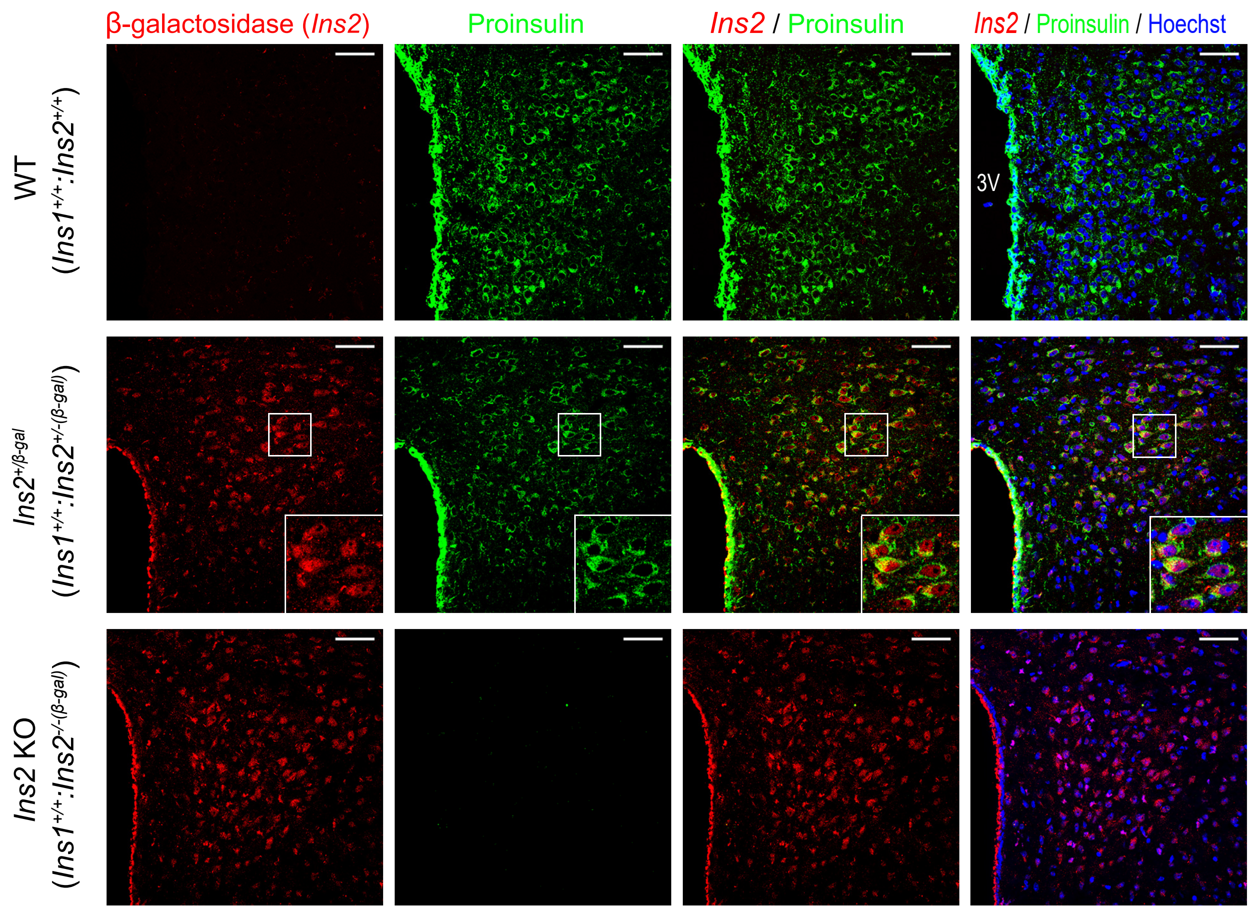

Supplemental Figure 3. Representative confocal images of double immunostaining for β-galactosidase (β-gal) and proinsulin in the PVN from WT, *Ins2*$^{+/β\text{-gal}}$, and *Ins2* KO mice.

Proinsulin protein in cell bodies (green) was co-localized with β-gal knocked into the endogenous *Ins2* locus (nucleus; red) in the PVN from *Ins2*$^{+/β\text{-gal}}$ mice (middle panel).

Scale bars: 50 μm.

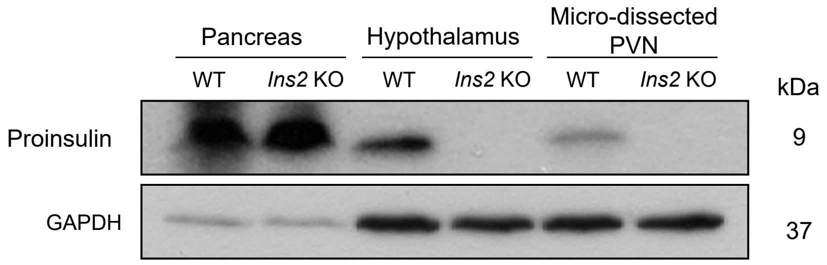

Supplemental Figure 4. Immunoblot analysis of proinsulin expression in the pancreas, hypothalamus, and micro--dissected PVN from WT and *Ins2* KO mice.

Proinsulin-positive band was still observed in *Ins2* KO pancreas as well as in WT pancreas, suggesting that proinsulin antibody (1:1000, Cell Signaling Technology, #8138) used in this analysis can detect not only mouse proinsulin 2 but also proinsulin 1 form. The antibody also yielded a positive band in the hypothalamus and micro-dissected PVN of WT mice, respectively, but none in those of *Ins2* KO mice.

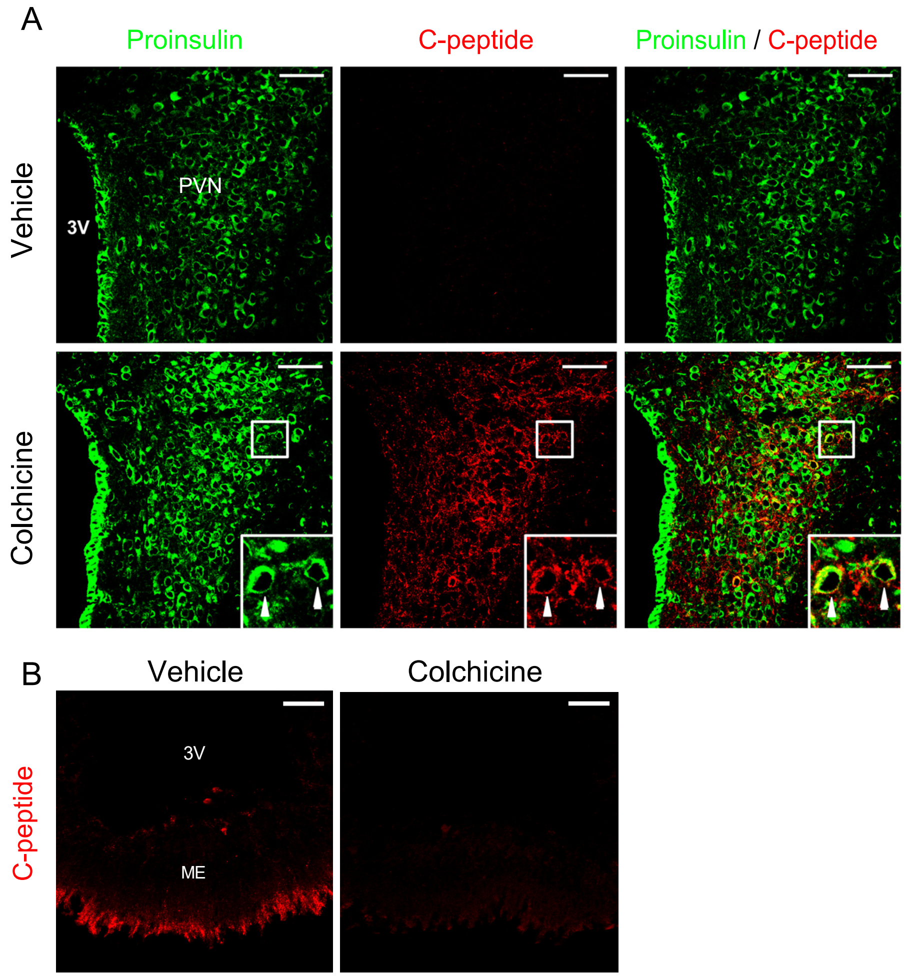

Supplemental Figure 5. Representative immunofluorescence images showing blockage of axonal transport of C-peptide by cholchicine injection.

(**A, B**) Mice were injected with vehicle or cholchicine into the left lateral ventricle. The PVN was immunostained for proinsulin and C-peptide (A) and the ME for C-peptide (B). Solid arrowheads show co-localization of proinsulin and C-peptide.

Scale bars: 50 µm.
